# Mental health and wellbeing during the transition to fatherhood: a systematic review of first time fathers’ experiences

**DOI:** 10.11124/JBISRIR-2017-003773

**Published:** 2018-11-15

**Authors:** Sharin Baldwin, Mary Malone, Jane Sandall, Debra Bick

**Affiliations:** 1 Florence Nightingale Faculty of Nursing, Midwifery and Palliative Care, King's College London, London, UK; 2 London North West University Healthcare NHS Trust, London, UK; 3 Department of Women and Children's Health, School of Life Course Sciences, Faculty of Life Sciences and Medicine, King's College London, London, UK; 4 The Nottingham Centre for Evidence-Based Healthcare: a Joanna Briggs Institute Centre of Excellence

**Keywords:** First time father, expectant father, mental health, wellbeing, perinatal period

## Abstract

**Objective:**

The aim of this systematic review was to identify and synthesize the best available evidence on first time fathers’ experiences and needs in relation to their mental health and wellbeing during their transition to fatherhood.

**Introduction:**

Men's mental health and wellbeing during their transition to fatherhood is an important public health issue that is currently under-researched from a qualitative perspective and poorly understood.

**Inclusion criteria:**

Resident first time fathers (biological and non-biological) of healthy babies born with no identified terminal or long-term conditions were included. The phenomena of interest were their experiences and needs in relation to mental health and wellbeing during their transition to fatherhood, from commencement of pregnancy until one year after birth. Studies based on qualitative data, including, but not limited to, designs within phenomenology, grounded theory, ethnography and action research were included.

**Methods:**

A three-step search strategy was used. The search strategy explored published and unpublished qualitative studies from 1960 to September 2017. All included studies were assessed by two independent reviewers and any disagreements were resolved by consensus or with a third reviewer. The recommended Joanna Briggs Institute (JBI) approach to critical appraisal, study selection, data extraction and data synthesis was used.

**Results:**

Twenty-two studies met the eligibility criteria and were included in the review, which were then assessed to be of moderate to high quality (scores 5–10) based on the JBI Critical Appraisal Checklist for Qualitative Research. The studies were published between 1990 and 2017, and all used qualitative methodologies to accomplish the overall aim of investigating the experiences of expectant or new fathers. Nine studies were from the UK, three from Sweden, three from Australia, two from Canada, two from the USA, one from Japan, one from Taiwan and one from Singapore. The total number of first time fathers included in the studies was 351. One hundred and forty-four findings were extracted from the included studies. Of these, 142 supported findings were aggregated into 23 categories and seven synthesized findings: 1) New fatherhood identity, 2) Competing challenges of new fatherhood, 3) Negative feelings and fears, 4) Stress and coping, 5) Lack of support, 6) What new fathers want, and 7) Positive aspects of fatherhood.

**Conclusions:**

Based on the synthesized findings, three main factors that affect first time fathers’ mental health and wellbeing during their transition to fatherhood were identified: the formation of the fatherhood identity, competing challenges of the new fatherhood role and negative feelings and fears relating to it. The role restrictions and changes in lifestyle often resulted in feelings of stress, for which fathers used denial or escape activities, such as smoking, working longer hours or listening to music, as coping techniques. Fathers wanted more guidance and support around the preparation for fatherhood, and partner relationship changes. Barriers to accessing support included lack of tailored information resources and acknowledgment from health professionals. Better preparation for fatherhood, and support for couple relationships during the transition to parenthood could facilitate better experiences for new fathers, and contribute to better adjustments and mental wellbeing in new fathers.

## ConQual Summary of Findings^1^

**Figure:**
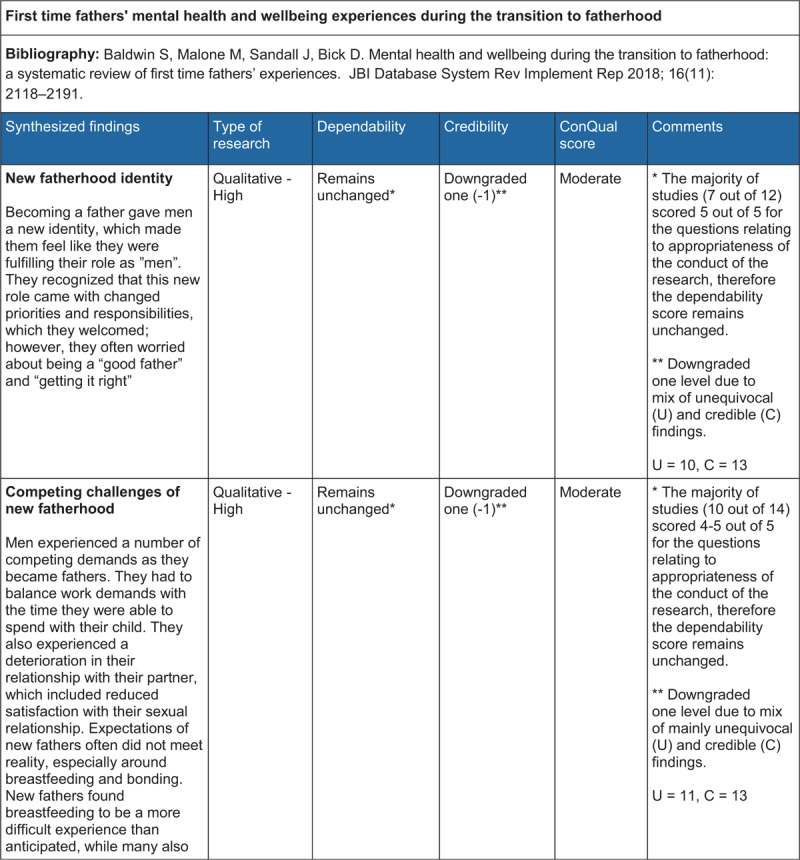


**Figure:**
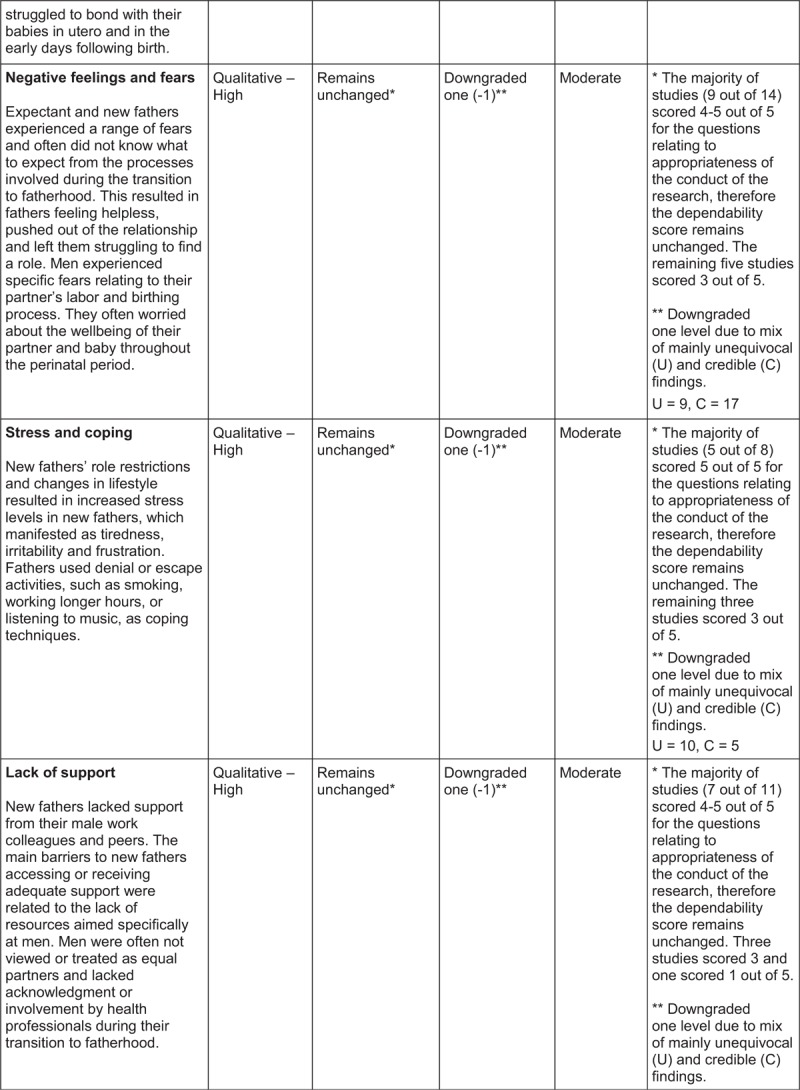


**Figure:**
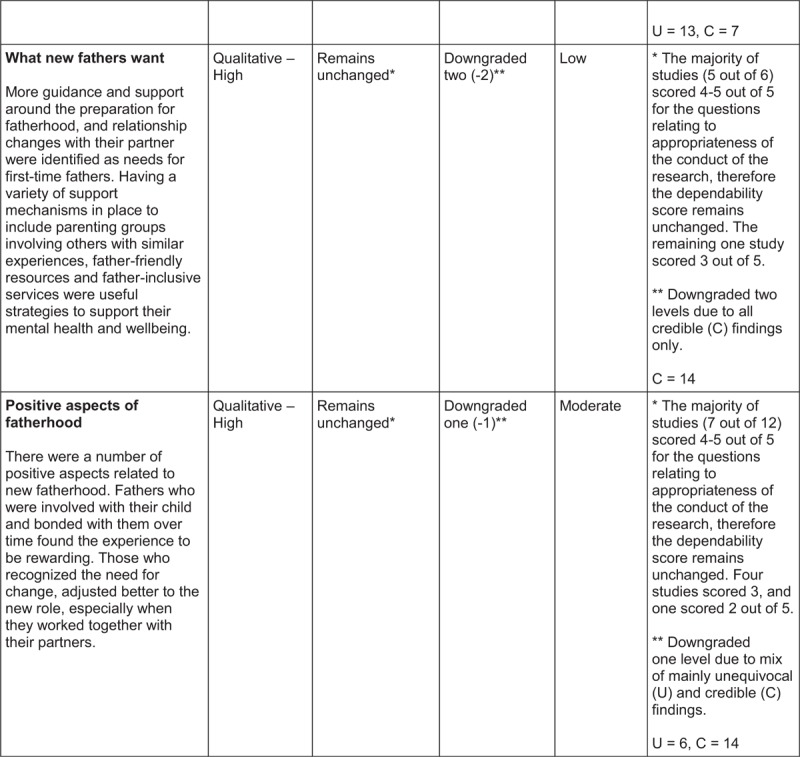


## Introduction

### Fathers’ mental health and wellbeing

The World Health Organization (WHO) defines mental health as “a state of wellbeing in which the individual realizes his or her own abilities, can cope with the normal stresses of life, can work productively and fruitfully, and is able to make a contribution to his or her community.^2^^(p.XIX)^ The Royal Society for Public Health in the UK has recommended that it is important to actively promote positive mental wellbeing rather than just focusing on preventing and treating mental illness.^3^ Men's mental health and wellbeing during their transition to fatherhood is an important public health issue that continues to be under-researched from a qualitative perspective and poorly understood.^4^ Poor mental health in fathers can impact negatively on their children, their partner and wider society.

Ramchandani *et al.*^5^ in a prospective cohort study, which controlled for mothers’ depression and fathers’ education levels, found that severe postnatal depression in fathers was associated with emotional and behavioral problems in their children at three years of age, particularly in boys. Moreover, children with two depressed parents were at higher risk of poor development outcomes.^6^ In a later study, Ramchandani *et al.*^7^ also reported an increased risk for psychiatric, behavioral and conduct disorders in children aged seven years if their fathers had been depressed during the postnatal period. Several studies have suggested a link between poor cognitive, behavioral, social and emotional development in children, and a negative father-child relationship.^8-12^ Poor mental health in fathers can also have an impact on the mother and the couple's relationship.^13^ A study of first time parents’ transition to parenthood highlighted the importance of focusing interventions on strengthening couple relationships and parents’ feelings of unworthiness.^14^

Anxiety and depression are the two most common mental health problems experienced by fathers in the perinatal period.^4,15-24^ A recent systematic review of 43 papers reported the prevalence rates of anxiety disorder in men to range between 4.1%–16% during their partners’ pregnancy and 2.4%–18% during the postnatal period.^15^ In another systematic review of 20 studies, the prevalence rates of antenatal and postnatal depression in fathers ranged from 1.2%–25.5%.^16^ With the exception of one study, which assessed depression through symptoms in a qualitative interview, the remaining studies in this review used standardized self-report instruments with established reliability and validity.^16^ A meta-analysis of 43 studies reported depression in 10.4% of fathers between the first trimester of their partner's pregnancy and one year postpartum, with the peak time being between three and six months after the birth, similar to findings for postnatal women.^4^ Symptoms of anxiety and stress have also been reported alongside depression among men during and after their partner's pregnancy.^17-23^ A literature review of 32 studies published between 1989–2008 on men's psychological transition to fatherhood, found pregnancy to be the most demanding period for the fathers’ psychological reorganisation of self, and labour and birth to be the most emotional moments involving highly mixed feelings, ranging from helplessness and anxiety to pleasure and pride.^24^ The postnatal period (defined in the review as up to one year following birth), however, was the most challenging time, due to fathers having to balance the various demands placed on them including personal and work related needs, their new role as a parent, emotional and relational needs of the family, and societal and economic pressures.^24^ A key element highlighted in the review was the importance of the quality of each man's relationship with his partner across the antenatal, intrapartum and postnatal periods. The study included resident fathers, but not non-biological fathers (such as adoptive fathers), stepfathers or fathers in same sex relationships. Therefore, the experiences of non-biological fathers during their transition to fatherhood remains unknown.

A more recent systematic review of 18 studies which examined stress in fathers in the perinatal period indicated that fathers’ stress levels increased from the antenatal period to the time of birth, with subsequent decrease in stress levels from birth to the later postnatal period,^23^ in contrast to the above findings.^24^ The main factors that contributed to stress in fathers in the perinatal period included negative feelings about the pregnancy, role restrictions related to becoming a father, fear of childbirth and feelings of incompetence about infant care.^23^

### Current interventions and gaps in evidence

A Cochrane systematic review of group-based parenting programs for improving parental psychosocial health, found that only four of the 48 included studies reported separate outcome data from fathers.^25^ While these showed a statistically significant short-term improvement in paternal stress following interventions that included cognitive and behavioral strategies, individual study results were inconclusive for any effect on depressive symptoms, confidence or partner satisfaction. The review authors concluded that this was “a serious omission given that fathers now play a significant role in childcare and research suggests that their psychosocial functioning is key to the wellbeing of children”.^25^^(p.21)^

A review of interventions for prevention or treatment of depression in fathers identified four studies, all focusing on treatment rather than prevention, and reported inconclusive findings due to wide study heterogeneity.^26^ The reviewers recommended the need for randomized controlled trials of effective mental health interventions for men in the postnatal period, particularly preventative interventions. Although this study was described as a systematic review, there was no evidence of the included studies being critically appraised, which raises concerns about the quality of the findings. Another systematic review of intervention programs to prevent or treat paternal mental illness in the perinatal period included 11 studies: five of which described psychosocial programs (emphasising skills, knowledge, emotional wellbeing, and social wellbeing related to parenting), three focused on the effects of massage techniques (partner massage and infant massage), and three which used couple-based sessions (focused on the couple relationship and co-parenting).^27^ Six of the eight randomized controlled trials included did not provide adequate information on randomisation processes and risk of bias could not be ruled out. The review authors reported significant intervention effects for a range of fathers’ mental health outcomes (including stress, depression, anxiety, anger levels and self-esteem) for two trials of psychosocial approaches,^28,29^ and three of massage techniques.^30-32^ There were no significant changes reported in paternal mental health following couple-based interventions.

Health professionals’ failure to engage with fathers during or around the time of birth could be a reason for the lack of evidence on first time fathers’ mental health and wellbeing.^33^ Fathers may feel marginalised and unacknowledged by health professionals during the perinatal period, and report a lack of appropriate information on pregnancy, birth, childcare, and balancing work and family responsibilities.^34-36^ Research into the role of health visitors (public health nurses in the UK) found that they do not involve fathers in routine contacts^37^ and were perceived by some fathers as a service provided “by women, for women”.^38^ A Department of Health for England funded literature review on service users’ views suggested that some fathers welcomed the opportunity to express their feelings and emotions about fatherhood when asked by a healthcare professional,^39^ but did not always have the opportunity to do this spontaneously.^40^

A systematic review of evidence on parenting interventions which included men as parents or co-parents showed that insufficient attention was paid to reporting fathers’ participation and fathers’ impacts on child or family outcomes.^41^ A rapid review to update evidence for the Healthy Child Programme in England included systematic review level evidence published from 2008 to 2014.^42^ It recognized the need to support fathers during the transition to parenthood, the lack of interventions designed specifically to support fathers and the need for further evaluations of parenting interventions that actively engaged fathers. The review made no specific reference to interventions aimed at improving fathers’ mental health and wellbeing during the perinatal period. This highlights the crucial importance of assessing men's mental health in the perinatal period,^43^ and identifying the best approaches to supporting fathers.^44^

While a number of studies relating to fathers’ mental health have been discussed above, the majority of the studies were quantitative in nature, focusing on incidence and symptoms. Few studies to date have explored first time fathers’ experiences and their perceived needs, or distinguished between biological and non-biological fathers, or if fathers were resident or non-resident in the family home. Better understanding of the experiences of first time fathers during their transition to fatherhood and identifying the level and content of information and support which could help their mental health and wellbeing, could inform the healthcare professional-led interventions acceptable to meet their needs. Barriers and facilitators to first time fathers accessing timely and appropriate support for their mental health and wellbeing needs could also be identified. This systematic review aimed to create a deeper knowledge of first time fathers’ experiences, needs and help seeking behaviors relating to mental health and wellbeing during their transition to fatherhood and how fathers could be better supported during this time.

In this review, first time fathers refers to men becoming either a biological or non-biological parent for the first time, and resident fathers refers to those who resided with their expectant partner, or their partner and child during their transition to fatherhood. The transition to fatherhood was defined as the period from conception to one year after birth. Mental health problems included any psychological difficulty or distress including depression, anxiety and stress. These may have been diagnosed by a health professional or self-reported by fathers. Mental wellbeing included positive mental health, covering both the hedonic (feeling good) and eudemonic components (functioning well) of psychological wellbeing.

Initial searches of the *JBI Database of Systematic Reviews and Implementation Reports*, Cochrane Library, MEDLINE, PROSPERO and DARE databases were conducted and although a small number of systematic reviews relating to this topic were identified and cited above, no qualitative systematic reviews were identified which answered the questions of this review.

The aim of this qualitative review was therefore to identify first time fathers’ experiences and needs in relation to their mental health and wellbeing during their transition to fatherhood.

## Review question/objective

The objective of this systematic review was to identify and synthesize the best available evidence on first time fathers’ experiences and needs in relation to their mental health and wellbeing during their transition to fatherhood.

Specifically, it sought to evaluate:

- How mental health and wellbeing are experienced by first time fathers.
- The perceived needs of first time fathers around mental health.
- The ways in which mental health problems are experienced, manifested, recognized and acted upon by first time fathers.
- The contexts and strategies that are perceived by first time fathers to support mental wellbeing.
- The perceived barriers and facilitators to first time fathers accessing support for their mental health and wellbeing.

## Inclusion criteria

### Participants

Study participants included first time fathers of healthy babies born at full term with no identified terminal or long-term conditions. As this review focused on the mental health and wellbeing of fathers in general and not of those with specific additional needs, studies were excluded if they considered:

- Non-resident/absent fathers (those not residing with the partner/child during the period between conception to one year after birth).
- Fathers experiencing bereavement following neonatal death, stillbirth, pregnancy loss or sudden infant death.
- Fathers whose infants were born prematurely (≤37 weeks gestation).
- Fathers of a child with terminal/long term conditions.

### Phenomena of interest

The phenomenon of interest for this review was first time fathers’ experiences and needs during their transition to fatherhood in relation to their mental health and wellbeing.

### Context

This review considered studies undertaken in high income countries as defined by the World Bank^45^ (for example, countries which are members of the European Economic Community, the UK, the USA, Canada, Australia and New Zealand). The majority of these countries have similar healthcare systems (with a mix of public and privately funded universal service provision), and social and political systems, meaning that review findings are likely to be more transferable.

### Types of studies

The review considered studies that focused on qualitative data including, but not limited to, designs such as phenomenology, grounded theory, ethnography and action research. The review also considered qualitative data reported within quantitative surveys for inclusion, where open questions relating to the phenomena of interest had been asked.

## Methods

The objectives, inclusion criteria and methods of analysis were specified in advance. The review was conducted according to the protocol, published in the *JBI Database of Systematic Reviews and Implementation Reports* (DOI: 10.11124/JBISRIR-2016-003031).^46^ The protocol was also registered with PROSPERO (PROSPERO 2016:CRD42016052685).

### Search strategy

The search strategy aimed to identify published and unpublished studies. A three-step search strategy was utilized. An initial limited search of MEDLINE (using Ovid) and CINAHL was undertaken followed by analysis of the text words contained in the title and abstract, and index terms used to describe the article. A second search using all identified keywords and index terms was then undertaken across all included databases. Thirdly, the reference list of all identified reports and articles were searched for additional studies.

Studies published in English were considered for inclusion as resources for translation were not available to the reviewers. Searches were conducted between January and September 2017 and computerized searches for studies published from 1960 to the present were considered for inclusion, to reflect the shift in fathers’ roles following the feminist movement.

The databases searched included: MEDLINE (Ovid), CINAHL, Embase, PsycINFO, Maternity and Infant Care, HMIC, British Nursing Index and Web of Science.

Searches were also carried out of the website of The Fatherhood Institute, the UK's leading charitable organisation for fathers and fatherhood. The Institute collates and publishes international research on fathers and impact of their role on children and mothers.

The search for unpublished studies such as theses and dissertations included: ProQuest Dissertations and Theses Global and WorldCat Dissertations and Theses (OCLC).

A full list of all databases searched and papers identified are presented in Appendix I, and an example of one of the searches undertaken (MEDLINE) is presented in Appendix ll.

### Study selection

The initial database searches and citation tracking was performed by the first author (SB). After pooling the retrieved titles, all duplicates were removed. Two reviewers (SB, DB) screened the titles independently and the final list of potential titles was created by compiling the lists of the two reviewers. The same process was repeated during the abstract screening where each reviewer read the abstracts independently and the selected abstracts were merged. Authors of the primary studies were contacted when the full text articles were not accessible. Discrepancies between the reviewers were resolved through comprehensive discussions to reach an agreement.

There were a number of studies where it was unclear if participants were first time or subsequent fathers, or residing with their partners or not. For such papers, where the authors’ contact details were available, they were contacted for further clarification. Papers were excluded if it was not possible to obtain further clarification. Quantitative studies, review articles, meta-analyses or meta-syntheses, editorials, commentaries, letters, conference abstracts, studies with no available full-text and non-English studies were also excluded.

### Assessment of methodological quality

Qualitative papers selected for retrieval were assessed by two independent reviewers (SB, DB) for methodological validity prior to inclusion in the review using the JBI Critical Appraisal Checklist for Qualitative Research as appended in the original protocol for this review.^46^ This enabled the reviewers to engage with and better understand the methodological strengths and limitations of the selected primary studies.

### Data extraction

Qualitative data were extracted from the included papers using the standardized JBI data extraction tool.^46^ The data extracted included specific details on methodology, methods, phenomena of interest and findings relevant to the review question and specific objectives. During the data extraction process, a level of “credibility” was allocated to each finding based on the degree of support offered by each illustration associated with it. The first reviewer assigned levels of credibility to each of the findings using the three levels as described by the standardized JBI qualitative data extraction tool:

i) Unequivocal (U): findings accompanied by an illustration beyond reasonable doubt and therefore not open to challenge.
ii) Credible (C): findings accompanied by an illustration lacking clear association with it and therefore open to challenge.
iii) Unsupported (US): findings are not supported by the data.

These were then discussed amongst the three reviewers (SB, DB, MM) resulting in general consensus with allocation of these levels.

### Data synthesis

Qualitative research findings were pooled using Joanna Briggs Institute System for the Unified Management, Assessment and Review of Information (JBI SUMARI). Findings were identified through repeated reading of text, and selection of themes from the results section. Most of the findings were based on the themes identified by the study authors in their qualitative analysis. The three-step process of data synthesis involved:

i) Extraction of all findings from all included papers with an accompanying illustration and establishing a level of credibility for each finding.
ii) Categorization of findings based on the similarity in meaning and concepts.
iii) Development of a comprehensive set of aggregated findings (of at least two categories) that could be used as a basis for evidence-based practice.

The categories, synthesized findings and accompanying descriptions were created using words and terminologies used by participants in the illustrations. These were discussed by the review team and revised until consensus was reached, prior to finalization. The reviewers also evaluated the synthesized findings with the ConQual^1^ approach to establish a level of confidence in each synthesized finding (Summary of Findings).

## Results

### Study inclusion

**Figure 1:**
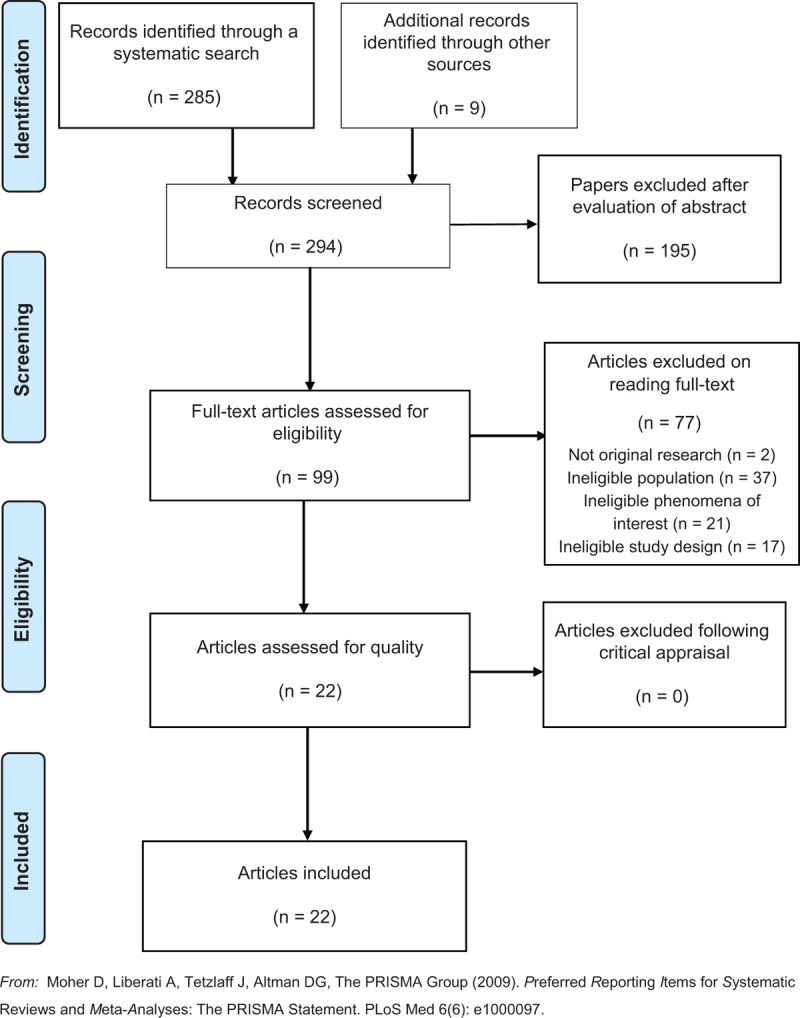
PRISMA flow diagram of search and study selection process

The literature search initially returned 285 records through database searching and nine through hand-searching reference lists of these papers, resulting in 294 potentially relevant records. On further examination of the study titles and abstracts, 195 of these records were excluded for various reasons, including duplication, quantitative study designs, opinion papers, those not focusing on the phenomena of interest or not meeting the inclusion criteria. The remaining 99 articles were retrieved for a full review, following which, 77 were excluded based on the agreed inclusion and exclusion criteria (two were not original research, 37 had ineligible population, 21 had ineligible phenomena of interest and 17 had ineligible study design), leaving 22 articles to be examined for methodological quality (see Figure 1).

### Methodological quality

The JBI Critical Appraisal Checklist for Qualitative Research^46^ provided a framework for scoring the quality of qualitative studies by addressing different aspects of the research such as ethical considerations, potential bias, integrity of the methodology, and congruity between methods, results and conclusion. Nine of the 22 studies scored 10 out of 10 on the JBI Critical Appraisal Checklist for Qualitative Research.^50,53,54,57,61,63,64,65,68^ Of the remaining studies, three scored nine,^49,59,60^ five scored eight,^47,51,52,58,66^ three scored seven,^55,56,67^ one scored six^48^ and one scored five.^62^ In all included studies there was congruity between the research methodology, research questions/objectives, and the representation and analysis of data. The descriptions of the methodology and methods of the 22 studies were clearly reported which supports the transferability of the findings. The analyses used for the studies were adequately described and were in line with the aims of the studies. On reviewing the papers, however, it was apparent that many of the studies did not include statements locating the researchers’ cultural or theoretical position, or the influence of the researcher on the research, and vice versa, making it difficult to determine the level of dependability of the study findings. This omission may have been due to the word restrictions set by journals. This was further discussed between the two reviewers (SB, DB) and for most of the papers any disagreements that arose between the two reviewers (SB, DB) were resolved through discussion. For two papers it was necessary to involve a third reviewer (MM). Following the third reviewer's (MM) appraisal of the papers and further discussion, consensus was reached among all three reviewers, which resulted in the final included papers presented in Table 1.

**Table 1:** Critical appraisal of included studies

Included studies	Q1	Q2^*^	Q3^*^	Q4^*^	Q5	Q6^*^	Q7^*^	Q8	Q9	Q10
Barclay and Lupton^47^	Y	Y	Y	Y	Y	Y	N	Y	N	Y
Bozlan *et al.*^48^	Y	Y	Y	Y	Y	N	N	Y	N	U
Dallos and Nokes^49^	Y	Y	Y	Y	Y	Y	Y	Y	N	Y
Darwin *et al.*^50^	Y	Y	Y	Y	Y	Y	Y	Y	Y	Y
Deave and Johnson^51^	Y	Y	Y	Y	Y	N	N	Y	Y	Y
De Montigny and Lacharité^52^	Y	Y	Y	Y	Y	N	N	Y	Y	Y
Dolan and Coe^53^	Y	Y	Y	Y	Y	Y	Y	Y	Y	Y
Finnbogadottir *et al.*^54^	Y	Y	Y	Y	Y	Y	Y	Y	Y	Y
Henderson and Brouse^55^	Y	Y	Y	Y	Y	N	N	Y	N	Y
Henwood and Procter^56^	Y	Y	Y	Y	Y	N	N	Y	U	Y
Ives^57^	Y	Y	Y	Y	Y	Y	Y	Y	Y	Y
Iwata^58^	Y	Y	Y	Y	Y	N	N	Y	Y	Y
Jordan^59^	Y	Y	Y	Y	Y	Y	Y	Y	N	Y
Kao and Long^60^	Y	Y	Y	Y	Y	Y	Y	Y	U	Y
Kowlessar *et al.*^61^	Y	Y	Y	Y	Y	Y	Y	Y	Y	Y
Machin^62^	U	Y	U	Y	Y	N	N	N	Y	Y
Olsson *et al.*^63^	Y	Y	Y	Y	Y	Y	Y	Y	Y	Y
Palsson *et al.*^64^	Y	Y	Y	Y	Y	Y	Y	Y	Y	Y
Poh *et al.*^65^	Y	Y	Y	Y	Y	Y	Y	Y	Y	Y
Rowe *et al.*^66^	Y	Y	Y	Y	Y	Y	N	Y	U	Y
Shirani and Henwood^67^	Y	Y	Y	Y	Y	N	U	Y	N	Y
Taniguchi *et al.*^68^	Y	Y	Y	Y	Y	Y	Y	Y	Y	Y

^*^ConQual dependability questions: N, No; U, Unclear; Y, Yes. Criteria for the critical appraisal of qualitative evidence: Q1 = Is there congruity between the stated philosophical perspective and the research methodology? Q2 = Is there congruity between the research methodology and the research question or objectives? Q3 = Is there congruity between the research methodology and the methods used to collect data? Q4 = Is there congruity between the research methodology and the representation and analysis of data? Q5 = Is there congruity between the research methodology and the interpretation of results? Q6 = Is there a statement locating the researcher culturally or theoretically? Q7 = Is the influence of the researcher on the research, and vice-versa, addressed? Q8 = Are participants, and their voices, adequately represented? Q9 = Is the research ethical according to current criteria or, for recent studies, and is there evidence of ethical approval by an appropriate body? Q10 = Do the conclusions drawn in the research report flow from the analysis, or interpretation, of the data?

The 22 included papers were assessed to be of moderate to high quality as the score ranged between 5 and 10 on the JBI Critical Appraisal Checklist for Qualitative Research and therefore none were excluded for reasons of quality. Table 1 includes assessments of methodological quality and corresponding results.

### Characteristics of included studies

The 22 included studies were published between 1990 and 2017, and all used qualitative methodologies to investigate the experiences of expectant or new fathers. Nine studies were from the UK, three from Sweden, three from Australia, two from Canada, two from the USA, one from Japan, one from Taiwan and one from Singapore. The total number of first time fathers included in the studies was 351.

For the 22 included qualitative papers:

- Methods included: phenomenology (seven), unspecified qualitative (eight), grounded theory (two), discourse analysis (two), narrative (two) and critical incident technique (one).
- Twenty studies focused on first time fathers only, investigating their expectations, experiences, views, needs or involvement as new fathers. Of these, two studies included couples and two included both expectant and new fathers.
- The remaining two studies included both first time and subsequent fathers, one specifically investigating experiences of paternal perinatal mental health and the other sexual relationship following birth from the perspective of male partners.
- The data collection methods used were primarily semi-structured or in-depth interviews, carried out face-to-face or by telephone. In two studies, group discussions/focus groups were used in addition to the interviews.
- Data analysis methods were consistent with the qualitative methodology used in each individual study.

Full characteristics of the 22 included studies are presented in Appendix III.

## Review findings

Each included paper was read by the two reviewers (SB, DB) and findings extracted. Each finding was accompanied by illustrations from the study to place them in context and assigned a level of credibility. For example, finding 3 was *Changing relationship with partner*. This was supported by an illustration from the study as follows:

“The first week was great, then after that things started to get worse. I never thought that Jenny and I would have fought so much”.^47^^(p.1018)^

This illustration supported the authors’ finding, and as risk of misinterpretation was minimal, it was considered to be “unequivocal”.

In total, 144 findings were identified and the same process was followed. Fifty-nine (41%) findings were unequivocal (U), 83 (58%) credible (C), and only two (1%) unsupported (US). As inclusion of unsupported findings is not recommended in JBI qualitative systematic reviews, the two unsupported findings were excluded from the next stages of the meta-synthesis. A full list of findings along with illustrations and levels of credibility are presented in Appendix IV. The 142 included findings were repeatedly read and reread to compare and identify similarities between them. Those found to be similar were aggregated into 23 categories as follows:

- Being a father, feeling more of a man
- Changed priorities, responsibility and expanded vision
- Being a good enough dad and getting it right
- Challenges of balancing work and the role of fatherhood
- Deterioration in couple relationship
- Changes to sexual relationship
- Breastfeeding: a difficult experience
- Struggles with bonding with the baby during pregnancy and the early days
- Not knowing what to expect and fear of the unknown
- Feelings of helplessness
- Pushed out of the relationship and struggling to find a role
- Fears relating to labor and birth
- Concerns about their partner's and baby's wellbeing
- Restrictions, frustrations and stresses of new fatherhood
- Coping mechanisms
- Societal expectations and lack of social/peer support
- Lack of tailored support or information resources for fathers
- Lack of acknowledgment and involvement by health professionals
- Need for guidance around preparing for fatherhood and relationship changes
- Preferred sources of information and support
- The rewards of bonding with their child
- Recognizing and adjusting to changes of parenthood
- Working in partnership.

A full list of findings and categories is presented in Appendix V. The categories were further examined to identify if they could be synthesized. Seven synthesized findings were identified:

1. New fatherhood identity
2. Competing challenges of new fatherhood
3. Negative feelings and fears
4. Stress and coping
5. Lack of support
6. What new fathers want
7. Positive aspects of fatherhood.

The ConQual^1^ approach to assess the confidence in the level of evidence of each synthesized finding was applied (Summary of Findings). For synthesized findings 1, 2, 3, 4, 5 and 7, the majority of the studies received four to five “yes” responses on the ConQual identified criteria for dependability; therefore, the level of confidence remained unchanged. The findings included a mix of unequivocal and equivocal (credible) ratings, necessitating downgrading by an additional level, resulting in a ConQual score of “moderate”. For synthesized finding 6, although the majority of studies (five out of six) scored four or five for the questions on appropriateness of research conduct (meaning no change to the dependability score), the credibility score was downgraded two levels (−2) due to all findings being equivocal (credible). The ConQual score for this finding was “low”. The seven synthesized findings are presented below and the relationship between study findings, categories and synthesized findings are illustrated in Tables 2–8.

**Table 2:** Synthesized finding 1: New fatherhood identity

Findings	Categories	Synthesized findings
What it means to be “male” [U] The perceived positive relationship between being male and the ability to father children [U] Feeling of development [U] The caring father might emerge as, in fact, the bigger bloke [C] Accomplishing an important goal in this life phase [C] Proving their ability as men [C]	Being a father, feeling more of a man	*New fatherhood identity:* Becoming a father gave men a new identity, which made them feel like they were fulfilling their role as “men”. They recognized that this new role came with changed priorities and responsibilities, which they welcomed; however, they often worried about being a “good father” and “getting it right”.
Maintaining health to meet the needs of forthcoming dependents [C] Feeling of responsibility [U] Symbolizing eternal love [C] Preparation for fatherhood [C] Expanded vision [U] Changes associated with the father's role [U] Adjusting priorities [C] Emotional changes experienced [C]	Changed priorities, responsibility and expanded vision
New fathers wish to father differently from their own fathers [U] Worry about being able to manage being both a good provider and a “hands on” father [U] Wanting to cherry pick the best bits from own childhood [C] Wanting to bring baby up in best way [C] Wanting to get things right [U] Worries about being a good enough dad [U] Expanded role of good fathers [C] Dealing with internal and external pressures [C] Good father and father involvement [C]	Being a good enough dad and getting it right

U, unequivocal; C, credible

**Table 3:** Synthesized finding 2: Competing challenges of new fatherhood

Findings	Categories	Synthesized findings
Renegotiating paid employment and household work or childcare work [U] Going to work/wanting to parent [U] Tensions and difficulties: cash and/or care? [C] Work life [U]	Challenges of balancing work and the role of fatherhood	*Competing challenges of new fatherhood:* Men experienced a number of competing demands as they became fathers. They had to balance work demands with the time they were able to spend with their child. They also experienced a deterioration in their relationship with their partner, which included reduced satisfaction with their sexual relationship. Expectations of new fathers often did not meet reality, especially around breastfeeding and bonding. New fathers found breastfeeding to be a more difficult experience than anticipated, while many also struggled to bond with their babies in utero and in the early days following birth.
Changing relationship with partner [U] Relationship deterioration [U] Maintaining conjugal functioning [C]	Deterioration in couple relationships
Societal view of sexuality [C] Expectations on sexuality in the relationship after childbirth [U] Changes in the relation after childbirth [C] Experience of sexual life after childbirth [C] Physical and mental alterations in partner [C]	Changes to sexual relationship
Coping with parental demands [U] Coming to terms with environmental demands [C] Breastfeeding: more challenging than expected [U]	Breastfeeding: a difficult experience
Expectations and symbolic meaning of fatherhood [U] Feeling of unreality [C] On the inside, looking in [U] Feeling like a father [C] Being aware of the difference between oneself and one's wife [U] Grappling with the reality of the pregnancy and child [C] Discouraged by the inapplicability of the old ways of building relationships [C] Experiences during pregnancy: Feelings of separation [C] Challenges in transition to parenthood [C]	Struggles with bonding with the baby during pregnancy and the early days

U, unequivocal; C, credible

**Table 4:** Synthesized finding 3: Negative feelings and fears

Findings	Categories	Synthesized findings
The birth [C] Fatherhood [C] Feeling of insufficiency and inadequacy [C] Expectations [U] A different mission and challenge [C] Challenges in pregnancy, childbirth, and parenting as husbands/partners [C]	Not knowing what to expect and fear of the unknown	*Negative feelings and fears:* Expectant and new fathers experienced a range of fears and often did not know what to expect from the processes involved during the transition to fatherhood. This resulted in fathers feeling helpless, pushed out of the relationship and struggling to find a role. Men experienced specific fears relating to their partner's labor and the birthing process. They often worried about the wellbeing of their partner and baby throughout the perinatal period.
Deference and support: a moral response [C] Plugging away at the role-making of involved fatherhood [U] Fatherhood – the early days: helplessness [U] Feelings of exclusion [C]	Feelings of helplessness
Excitement thwarted by partner's reticence [C] The focus shifting from us to him [U] Feeling left/pushed out [U] Struggling to find a role [U] Apprehension about criticism [C] Helping out or “full involvement”? Fairness, equity and decision making [U]	Pushed out of the relationship and struggling to find a role
Aspects of the labor and birth [U] “Being there”: men's experiences of the labor and birth – cesarean [U]	Fears relating to labor and birth
Childbirth perceived as a shared experience and being there [C] Realizing oneself as a husband [C] Finding the wife's pregnancy and delivery for the first time to be an impressive experience [C] Ending their wives’ discomfort [C] The health status of his wife and fetus [C] The wonder of fetal movement [C] Imagining life and needs with a baby: fantasies and fears [C] Making active efforts in preparation for childbirth in a foreign country [C]	Concerns about their partner's and baby's wellbeing

U, unequivocal; C, credible

**Table 5:** Synthesized finding 4: Stress and coping

Findings	Categories	Synthesized findings
Challenges of combining new fatherhood and traditional narratives [U] Life's restrictions on becoming a parent [U] Articulating and attributing stress [U] Protecting the partnership [U] Coming to terms with the physical and emotional changes during the postpartum period [U] Whose needs? Whose values? Selflessness and autonomy in dialogue [C] Being tired and bound [C] Understanding emotional reactions [C]	Restrictions, frustrations and stresses of new fatherhood	*Stress and coping:* The role restrictions and changes in lifestyle resulted in increased stress levels in new fathers, which manifested as tiredness, irritability and frustration. Fathers used denial or escape activities, such as smoking, working longer hours or listening to music as coping techniques.
Engaging with traditional fatherhood [U] Not engaging with fatherhood [U] What is expected of men is different to how I feel! [C] Legitimacy of paternal stress and entitlement to health professionals’ support: Symptoms and manifestation [U] Managing stress through distraction, denial and release [U] Disclosing personal difficulties [U] Adjustment [C]	Signs of stress and coping mechanisms

U, unequivocal; C, credible

**Table 6:** Synthesized finding 5: Lack of support

Findings	Categories	Synthesized findings
Male friends at work unable to offer support [C] Social support [U] Feeling of social changes [U] Struggling for recognition as a parent from mate, co-workers, friends, family, baby and society [U] Government and society [U]	Societal expectations and lack of social/peer support	*Lack of support:* New fathers lacked support from their male work colleagues and peers. The main barriers to new fathers accessing or receiving adequate support were related to the lack of resources aimed specifically at men. Men were often not viewed or treated as equal partners and lacked acknowledgment or involvement by health professionals during their transition to fatherhood.
Lack of guidance and obstacles for achieving new fatherhood [U] Diversity of men's support networks: lack of information resources tailored to men [C] Information [C] Support [U] Lack of knowledge about childbirth [U] Experience of the NHS and father's wellbeing [U]	Lack of tailored support or information resources for fathers
Determination and sustained effort required to challenge the constructions of fatherhood [U] Entitlement to health professionals’ support [U] Involvement in healthcare provision [C] Self and other interacting with nurses: exchanging information with nurses [U] “Being there”: men's experiences of the labor and birth – presence during labor [C] “Being there”: men's experiences of the labor and birth – healthcare professional [C] Feeling of exclusion [U] Present, but not participating [U] Imagining life and needs with a baby: gendered roles [C]	Lack of acknowledgment and involvement by health professionals

U, unequivocal; C, credible; NHS, National Health Service

**Table 7:** Synthesized finding 6: What new fathers want

Findings	Categories	Synthesized findings
“Formal” peer support and opportunities to meet other fathers [C] Preparation for fatherhood [C] Parents’ relationships [C] Acknowledging ones’ limitations [C] The need for guidance [C]	Need for guidance around preparing for fatherhood and relationship changes	*What new fathers want:* More guidance and support around the preparation for fatherhood, and relationship changes with their partner were identified as needs for first-time fathers. Having a variety of support mechanisms in place to include parenting groups involving others with similar experiences, father-friendly resources and father-inclusive services were useful strategies to support their mental health and wellbeing.
Pre-existing networks – friends, family and the wider community [C] Parental groups: the good and the bad [C] Internet as an asset or a worrier [C] Information: the when and how [C] Social support received [C] Suggestions for improvement to the current maternity care [C] Preferred sources of information and support [C] The role of primary care in mental health care for new parents: routine enquiry [C] The role of primary care in mental health care for new parents: screening questionnaires [C]	Preferred sources of information and support

C, credible

**Table 8:** Synthesized finding 7: Positive aspects of fatherhood

Findings	Categories	Synthesized findings
Navigating fatherhood: Strength through fatherhood as rewarding [U] Feeling of reality [C] Transition to mastery [C] The pleasures, benefits and rewards of bonding with their child [U] Sharing time and space with one's child [C] Engagement [U] Fatherhood – the early days: gaining confidence and regaining control [C] Bonding and co-parenting [U]	The rewards of bonding with their child	*Positive aspects of fatherhood:* There were a number of positive aspects related to new fatherhood. Fathers who were involved with their child and bonded with them over time found the experience to be rewarding. Those who recognized the need for change, adjusted better to the new role, especially when they worked together with their partners.
Reality [C] Being aware of a change and trying to adjust to a new life [U] Fatherhood – the early days: trial and error parenting [U] Caring for the baby in both health and illness [C] Still being a couple but not as before [C] Imagining life and needs with a baby: relationships [C]	Recognizing and adjusting to changes of parenthood
Feeling prepared and (changing) expectations [C] Fatherhood – the early days: she leads, I follow [C] Fatherhood – the early days: working together [C] Communicating with ones’ partner [C] Forming a fatherhood identity [C] Adaptive and supportive behaviors adopted [C]	Working in partnership

C, credible; U, unequivocal

### Synthesized finding 1: New fatherhood identity

Three categories comprising 23 findings were integrated into the first synthesized finding (Table 2). The first category, *being a father, feeling more of a man,* refers to how men perceived themselves as they became fathers for the first time. Their ability to father a child was described as an important achievement in their lives: “My first thought was ‘yes! I can have a baby”’.^60^^(p.63)^

Becoming a father also made them feel more masculine and “more of a man”. One father described feeling “over the moon…I suppose it's like a man thing. It's like you feel more of a man in a way. I know it sounds a bit weird but you feel more a man…You feel everything's working and you’re alright. So I was over the moon, overjoyed”.^53^^(p.1023)^ While another father talked about development and growth resulting from new fatherhood: “I feel, that I’m growing, as a human being. Yes, it's what I’m doing, absolutely. And even as a man. That it's undeniably one kind of confirmation”.^54^^(p.102)^

The second category, *changed priorities, responsibility and expanded vision,* refers to how men acknowledged that the new role of fatherhood led to new responsibilities and priorities. Men talked about the need to change their lifestyle due to having “another person to think about”.

“You’ve got to leave your juvenile life behind, stop running around with your mates and that. You have to change it… without agreeing to it… you’ve just got no choice (laughs). Now I’ve got someone else to think about…”.^64^^(p.89)^

The changes also included taking on the provider role to ensure financial security: “ …there was something in the breadwinner factor that made me feel that I should change my priorities. It happens even before the baby is born. We are building our ‘nest’ and making more rational decisions than before”.^54^^(p.101)^ “Money is also very important. We therefore have to save as much as we can. I need to work as hard as possible. Maybe I’ll need some investments as well”.^60^^(p.66)^ Most men, however, welcomed these changes and felt were necessary for this new phase in their lives.

The third category, b*eing a good enough dad and getting it right,* refers to how men wanted to be a good father and often worried about “not getting it right”. Some men wanted to be more “hands on” with their child and father them differently to how they themselves were fathered: “My father was more removed, I’m much more hands on, my father sat around and did little, my experience is very different, I change nappies, make milk and get up in the middle of the night.”^48^^(p.74)^

Being a good father was seen as being present and spending time with their child: “I think being there for all their first major things is important, i.e. when they’re at school, when they go to do a nativity play, going to the nativity play, not saying no I’m too busy at work or, you know, someone will video it for me, or whatever.^56^^(p.343)^

“A parent who is prepared to put work second and family first, you know, the father who's prepared to do that, I think that's a good father.”^56^^(p.343)^

However, men often worried about not “getting things right” and not being able to fulfill the role of a good father: “I am also worried of not getting it right. Uh… do I let him play on the floor with the baby gym with all the things hanging all over the top; he's interested in that. But do I, do I leave him or not? Do… er… is that not interacting with him enough? But then, if I put him in the cot in his springy seat thing, but what am I supposed to say to him? Am I supposed just to play with him? Cuddle him? Am I supposed to? And… and I don’t naturally sort of feel, I don’t know what to do”^49^^(p.156)^

The synthesized finding summarizes how the transition to fatherhood is perceived by men. During this time a new fatherhood identity is formed, which makes them feel more masculine while accomplishing an important new phase in life.

### Synthesized finding 2: Competing challenges of new fatherhood

This meta-synthesis resulted from five categories, comprising 24 findings (Table 3). The first category, *challenges of balancing work and the role of fatherhood*, refers to the dilemma men experience as a result of having to balance their work responsibilities with being a father.

“I feel as though my work, because my family's number one my work's got to be number one at the moment and it's that, it's that absolutely what seems to be an irreconcilable tension between the fact that you work, you are working for your family and you’re trying to build a career. Because you know you want to spend, you’re trying to build a career because you want the time and the quality time to spend at home. And you’re building a career and as a result you’re not getting that quality time to spend at home. So you’re wanting both and if you don’t have one you haven’t got the other half, you know its um its really frustrating”^56^^(p.346)^

Men particularly worried about “missing out” on spending time with their child, because of work responsibilities: “I hope I’m around in those times when he is learning to play. There is a couple of hours each day when he wants to play and try and talk and stuff. Because I’m at work I hope I don’t miss out on that too much. I don’t want to come home all the time and [find] him asleep”.^47^^(p.1015)^

“After this last week away and seeing him grow and then going back to work and having 15 minutes a day with him… it has made me realize what I am missing and it is hard because you want to be there and you want to see everything… [The bond] has developed but because I don’t get to see him as often as I would like it is a constant worry that it is not developing how I would want it to…”^62^^(p.50)^

The second category was *deterioration in couple relationship* following the birth of their child. Men reported changes in their relationship with their partner, in some cases needing additional relationship interventions following the birth of their child: “The first week was great, then after that things started to get worse. I never thought that Jenny and I would have fought so much”.^47^^(p.1018)^

“Our relationship between the two of us has deteriorated quite drastically now. We are actually going to see Relate…. We go to Relate, we’ve been to Relate twice because Esme suggested we’d better go to Relate because we were, really we were, our relationship is not touching, not talking, nothing, nothing”^49^^(p.153)^

There were also *changes to sexual relationship* between couples following childbirth, which formed the third category. While this category resulted from five different findings, they derived from the same study. Generally, the changes referred to a deterioration in sexual activity: “Prior to the birth you think, ‘a few weeks abstinence,’ but now when the child is born… it can be half a year.”^63^^(p.720)^

Sex was also seen as less of a priority to women than men: “Altogether, sexual life is important in a relationship. To ‘K’ it comes far down on the priority list. To sleep 10 hours during the night, cleaning the house, doing the laundry and… when all this is done she can start thinking about having sex”.^63^^(p.721)^

The fourth category, *breastfeeding: a difficult experience*, refers to the challenges experienced by new fathers relating specifically to breastfeeding. It was something that fathers found difficult, anxiety provoking and that they were totally unprepared for: “It was one feeding after another; I was under the impression of having no respite. I knew it would be like that, but I still found it difficult.”^52^^(p.333)^

“I have to say that there I was not prepared at all but had a mental picture that it's just a matter of laying the baby to the breast and it all works. When it didn’t work you stood there: aha, what the hell do we do now?”^64^^(p.88)^

Fathers reported the experience to be more challenging than they had anticipated, which left them feeling “helpless”: “Breastfeeding was what I found most difficult. I didn’t know how to help, I felt useless.”^52^^(p.333)^

The fifth category was *struggles with bonding with the baby during pregnancy and the early days*. During the pregnancy period, men talked about not “feeling like a father” straightaway: “I don’t feel myself as a father, or how should I put it… I don’t feel it consciously. It was not like going up stairs and at a certain point, ‘I’m a father from today!’ Such a feeling didn’t come to me. It was more like going up a slope”.^58^^(p.162)^

They struggled to bond with their unborn child as the baby was not growing inside them, “ …My wife can share her feelings with me. Sometimes she says the baby is moving inside her. But, actually, as a third person, I can’t imagine what that's like”.^60^^(p.65)^

The struggles to bond with their baby continued after the birth. Many expected that they would bond immediately with the child and were surprised when that was not the case: “I thought as a father there would be a bond there straight away with the child. I thought it would just come naturally. I thought because he was mine I was going to be immediately attracted to this child and love would just come naturally. I was surprised I wasn’t overcome with feelings for him straight away”^47^^(p.1017)^.

There appeared to be a level of disappointment when the fathers’ expectations about bonding with their baby did not meet reality. Many fathers felt that the bond between the mother and child was much stronger.

Therefore, the current synthesized finding summarizes the various challenges experienced by men during their transition to fatherhood.

### Synthesized finding 3: Negative feelings and fears

This meta-synthesis resulted from five categories, with 26 findings (Table 4). The first category, *not knowing what to expect and fear of the unknown*, relates to men's expectations of labor, birth and the new father role. Men talked about feeling nervous and unprepared due to not having any previous experience: “I don’t know how to interact with my child when she's born… I’ve never been a father, so I feel quite terrified”.^60^^(p.64)^ They often did not know what to expect, “It's like hitting a brick wall It's like, when they put something up, you know it's going to be there but until you actually get there you don’t know what to expect”.^55^^(p.296)^

This uncertainty often resulted in men feeling frustrated, excluded and uncertain about how they could help, which formed the second category of *feelings of helplessness*: “You’re not overly sure what you’re supposed to be doing, and there are times when you have the emotion of complete helplessness.”^61^^(p.6)^

“Um things that I find difficult is not being able to stop that, not being able to stop her crying… That's hard because I feel quite helpless you know when she is really screaming her head off. Then Tanya usually has to breastfeed her or sometimes she just likes to nurse on Tanya, on Tanya's breast just to fall off to sleep sort of thing. So that is difficult not being able to do anything about that, I can’t feed her but I can’t do anything”.^67^^(p.21)^

The third category was *pushed out of the relationship and struggling to find a role*. Men described not feeling involved with their partner's pregnancy and the birth due to not being able to physically experience the changes: “And I felt really out of the whole thing… I wasn’t involved in that (the pregnancy)… I couldn’t be because it wasn’t in me… and all I could do was be there for her”.^49^^(p.155)^ Fathers wanted to be involved in decision making processes but were often left excluded, struggling to find a role.

The fourth category, *fears relating to labor and birth*, referred to the specific concerns expressed by men relating to the birthing process. All three findings in this category were derived from the same study. While men wanted to support their partner through labor and birth, they were often concerned about their ability to deal with it: “First and foremost I hope I don’t pass out. Because I don’t like needles and all that sort of stuff… It just sends me a bit funny… I’m hoping I won’t pass out anyway. But you never know”.^53^^(p.1025)^

The fifth category related to men's *concerns about their partner's and baby's wellbeing*. Men worried about their partner and baby during pregnancy, birth and the early days: “I would like to say that soon my wife will not be suffering any longer. She's been through a hard time; before she became pregnant, and now, while she is expecting this baby. As far as I know, she has gone through many hurdles such as examinations and extracting her legs. I’m not even sure if I could do the whole thing once and she tried many times. So, she is a great woman… Now it's successful and she’ll never have to go through any more suffering!”^60^^(p.63)^

All five categories in this synthesized finding related to negative feelings and fears experienced by men during their transition to fatherhood.

### Synthesized finding 4: Stress and coping

This meta-synthesis resulted from two categories, comprising 15 findings (Table 5). The first category was *restrictions, frustrations and stresses of new fatherhood*. Many fathers acknowledged the restrictions of their new role and not being able to do all the things that they wanted to do, which often led to frustration.

“One of the feelings I have been getting is of… I can’t do all the things I want to do. I found it very frustrating… I’ve been on leave for quite a lot recently… I find it very frustrating when I can’t, I can’t get to go and do something I want to do like… like the washing… something simple like that”.^49^^(p.158)^

“Um… I didn’t quite understand, I don’t think I quite understood how full on babies are. Er… they’re 100% and more. They take over your life and there's no… you don’t have a life in effect really”.^49^^(p.158)^

New fathers experienced tiredness, sleeplessness, exhaustion and irritation,^52,56,65^ which increased their stress levels in the postnatal period. One father described stress as the “non-stop-ness of it”^50^^(p.5)^ due to having a stressful job and no time to relax.

*Signs of stress and coping mechanisms* was the second category. Fathers talked about feeling grumpy and snappy as a result of the tiredness and stress, which they often managed through distraction techniques, such as getting engrossed in work, listening to music or smoking. “I’m probably the sort of bloke who actually just says, ‘oh I’m quite forgetful, so I can forget I’ve had the worst night ever’. I just try and forget it. So that's probably my coping mechanism. It's just, trying to forget it and I generally do. And then, I guess, I’ve found in some ways, work quite helpful in that respect, because you can have a crazy night where you have no idea what's going on with [son's name], but I can go to work and I feel fine. I’m in control here, I know what to do. There's people who I can actually communicate with, they’ll do what I ask them to do and vice versa. So I’m probably not the best example, the best person to ask, because I think I just choose to ignore. I’m probably more of an ignorer, which isn’t probably that helpful for [partner's name].”^50^^(p.8)^

“… She often complains that I download ‘noise’ from the internet. She thinks it's not music. I feel bad when she keeps going on at me about this. I just go outside and have a smoke”.^60^^(p.65)^

### Synthesized finding 5: Lack of support

This meta-synthesis was derived from 20 findings and three categories*: societal expectations and lack of social/peer support, lack of tailored support or information resources for fathers,* and *lack of acknowledgment and involvement by health professionals* (Table 6).

Many men talked about the lack of understanding from male friends and work colleagues about the challenges associated with their new role as fathers. They described not finding “anybody that is real understanding”,^59^^(p.14)^ feeling they had “drifted incredibly far apart” from friends^54^^(p.101)^ and how peers “just take the mickey really keep telling me my life as I know it is over”.^53^^(p.1026)^

The lack of tailored support and information resources for fathers was apparent. Men were unaware of resources designed specifically for “dads”, and felt services were mainly aimed at women. Many felt excluded by health professionals and described feeling like a “spare part”^50^^(p.10)^ and made “out to be a complete idiot”.^66^^(p.49)^ They were often not acknowledged as equal partners in the process as health professionals mainly focused on the mother. Many men, however, accepted this, as they felt their partners’ needs should be prioritized when healthcare resources are limited.

### Synthesized finding 6: What new fathers want

Two categories including 14 findings were integrated into the sixth synthesized finding (Table 7). The first category, *need for guidance around preparing for fatherhood and relationship changes*, refers to the identified perceived needs of first time fathers. They wanted practical advice around clothing, feeding and routines for the baby, as well as information around changes in their relationship with their partner following the birth of the baby, including sexual relationships.

“I would look now to wanting more information about what to do when I’ve actually got it… even little things like what clothing, when you put it to bed, getting into a routine, even the basics, really”.^51^^(p.630)^

“You are both tired, niggling at each other, and it was probably slightly worse from what we thought. I mean, if the awareness could have been made a lot more, because no one ever really spoke to us about that other side…the relationship with us and the baby. We sort of sat down and we tried about two or three different ways and thought about this”.^51^^(p.631)^

“The midwife was very nice… and she asked: do you have any questions? But you don’t have any questions if you don’t know what is coming. I would know now (after birth) what to ask”.^64^^(p.90)^

Fathers identified a number of different sources of information and support that would be helpful, which formed the second category, *preferred sources of information and support*. Many valued face to face contact, where information relating to the transition to parenthood was provided by a professional but felt that a variety of methods should be available to fathers.

“I learn most when someone tells me things… absolutely. So, I prefer that. But it's probably that you need to have a mixture of things… because some learn by reading and seeing”.^64^^(p.90)^

Others talked about having access to parenting groups or DVDs involving other parents, with similar experiences: “Seeing [on the DVD] not the specialists, not the experts but the guys who were actually going through that situation without knowing much, the way we do. I could identify with those”.^66^^(p.50)^

One father talked about the importance of making the information fun and humorous to capture their interest, while another talked about the dilemmas of using the internet due to not knowing how credible the information is: “Information needs to be well choreographed, it needs to capture our interest, it needs to be given in a fun way. Use humor: situations can afterwards be looked at as funny or comic but when you are in it, it's like a matter of life or death”.^64^^(p.90)^

“I looked at YouTube, but you don’t know to a hundred percent which… what experience those showing the film have…Yes, if you think a bit… is it something good or can it be harmful… ”^64^^(p.90)^

Family members, parents and parents-in-law, were seen as good sources of support, where available. Routine enquiry about emotional wellbeing, however, was questioned, as fathers were uncertain about their primary care professionals’ training around emotional wellbeing and ability to provide adequate support. In regard to screening questionnaires, men's willingness to complete them would depend on how long the form was, how they were feeling at the time, their perceived value of completing it at the time and if there were competing priorities.

### Synthesized finding 7: Positive aspects of fatherhood

This meta-synthesis resulted from three categories of 20 findings on positive aspects of transition to fatherhood (Table 8). The first category related to *the rewards of bonding with their child*. The more time men spent with their children, the more confident they felt as fathers, and they reported the experience to be extremely rewarding: “The sleepless nights do take their toll on you, but I don’t know if it's just the way that I think… but I tend to look at the bigger picture. I just think I’m happy because she's healthy, she's smiling… So I think, well, I must be doing something half right for her to be trotting around as she does, and she's happy with me”.^50^^(p.9)^

“I think the nicest bit is just spending time sitting around on the bed and just playing with him, and just talking to him and being talked back at, and changing his nappy when that happens as well and, you know, time looking at him and him looking at me really is the bit that I’m really enjoying”.^56^^(p.344)^

The second category, *recognizing and adjusting to changes of parenthood,* refers to fathers who recognized and accepted the changes to their lifestyles. They also appeared to make better adjustments to the new role of fatherhood: “Talking about meals, if at restaurants, I’m afraid that my daughter will cry to bother people, so I come to think of eating at home. I think our eating style has changed. But for me, it's not something inconvenient, unpleasant, nor restricted. Rather, I am enjoying the time”.^58^^(p.163)^“Initially it is all about trial and error, at least that's how it was for us, purely trial and error… in the early days we were both sort of saying, what's wrong with him? Is it his nappy? Is it food? Is it sleep? And you go through that sort of list until you find something that makes him quiet and you go, well it was that then, and so you start to notice those signs a little more each time”.^61^^(p.6)^

The third category, *working in partnership*, refers to couples who communicated well and worked together to address the challenges of parenthood.^50,61,65,66^

“Another thing we did was the both of us were getting up in the night to deal with [our daughter] and we soon realized that maybe I needed some more sleep so Anna [wife] would get up and do all the night feeds one night and I would do all the night feeds the next night… we soon got her onto the bottle so I could help out with the dream feeds while Anna slept and when she got up to do the next feed I would be able to go to sleep… working in partnership is key”.^61^^(p.6)^

“We’ve talked… through the whole pregnancy because things can change – what you think and believe. That way you avoid irritation and rows”.^64^^(p.89)^

## Discussion

This qualitative systematic review aimed to explore the experiences and needs of first time fathers in relation to their mental health and wellbeing during the transition to fatherhood. Twenty-two papers were included in the review after a rigorous search and inclusion process. While all included papers focused on the general experiences of expectant or new fathers, only three specifically addressed the mental health and wellbeing of first time fathers. Dallos and Nokes^49^ explored the experience of a first time father who encountered psychological difficulties following the birth of their baby; Darwin *et al.*^50^ looked at fathers’ views and experiences of paternal perinatal mental health; and Rowe *et al.*^66^ investigated first time expectant couples’ anticipated needs and preferred sources of mental health information and support. The remaining papers, although focused on general experiences of first time fathers did report on factors that affected their mental health and wellbeing in line with the review objectives.

All included papers were of moderate to high quality (scores 5–10) based on the JBI Critical Appraisal Checklist for Qualitative Research. However, when ConQual criteria^1^ determining dependability were considered in conjunction with criteria determining credibility, the level of evidence for six of the synthesized findings were rated as moderate, and one synthesized finding was rated as low. This discussion will examine each synthesized finding and consider implications for practice and further research.

The synthesized findings described men's experiences of first time fatherhood characterized by the formation of fatherhood identity, the competing challenges of their new role and the negative feelings and fears arising from the changes. For many new fathers the transition to fatherhood was the ”best experience” in their lives.^65^ The ability to father a child made men feel like they were accomplishing an important phase in their lives,^54,60^ which made them feel more masculine and “more of a man”.^53,56^ While their new role came with additional responsibilities, it gave men an expanded vision for the future.^53,54,60,64^ In addition, most men wanted to be good fathers and worried about “not getting it right”. The concept of “good fathering” was linked to their ability to financially provide for their child, supporting previous study findings where fathers viewed their financial duty as part of their identity and self-worth.^69^

The additional responsibilities and pressures to be a “good father” and meet expectations as a “father” and a “man” impact on men's mental health and wellbeing, particularly as they become a father for the first time.^26^ The current review found that men faced competing challenges during their transition to fatherhood and worried about “missing out” on moments with their child due to work demands and responsibilities.^47,49,56^ This is similar to the findings of a literature review in which fathers were reported to find the year following their child's birth particularly challenging due to the conflicting needs to balance personal and work-related necessities with their new role as a parent, meet emotional and relational needs of their family, and deal with societal and economic pressures.^24^

An important finding of the current review was that many men experienced a deterioration in their relationship with their partner following the birth of their child,^47,49,52^ including changes in their sexual relationships.^64^ This is not uncommon as the reduction in positive communication between couples following birth has been linked to a decline in relationship and marital satisfaction as well as an increase in conflict.^70,71,72,73,74^ In a study by Darwin *et al.*^50^, new fathers’ lack of sleep and emotional exhaustion led to increased levels of stress, which also impacted negatively on couple relationships as couples spent less time together and received less emotional support from one another. If relationships between couples following the birth of their child are fraught, postnatal depression may be more likely to develop in both parents in the first year of birth.^75^ Poor couple relationships and satisfaction are risk factors that have previously been associated with anxiety and depression in men during and following the period of transition to fatherhood.^12,13,35,36^ Although men talked about changes to their sexual relationships with their partner following the birth of their child,^63^ this was not necessarily perceived as a negative aspect. However, findings from this review show that new fathers would have preferred to know about some these possible challenges before the birth, so that they could be prepared for such relationship changes.

Another challenge experienced by new fathers in the review was related to breastfeeding. It was something that fathers found anxiety provoking and that they were totally unprepared for in terms of how to support their partner.^53,65^ Fathers reported experiences to be more challenging than they had anticipated, which left them feeling “helpless”. This suggests that fathers need appropriate information about breastfeeding prior to the birth of their baby and planned, ongoing support following the birth to ensure that they are well informed and can better support their partners. These findings were consistent with previous research which suggests that the attributes of positive father support in relation to breastfeeding is dependent on the father's knowledge about breastfeeding, their attitudes to breastfeeding, their involvement in the decision-making process about breastfeeding and their ability to provide practical and emotional support to their partner.^76^ There are a number of other benefits in providing fathers with this support: a woman's decision to breastfeed is often influenced by her partner's attitudes and behaviors towards breastfeeding;^77^ women feel more confident and capable about breastfeeding when their partner is supportive and involved, and breastfeeding is likely to be more successful.^78^ Successful breastfeeding also has the potential to positively influence the relationship between the parents.^77^

During the antenatal period, men described not “feeling like a father” straightaway^58^^(p.162)^ and struggled to bond with their unborn child.^60^^(p.65)^ Their struggles to bond with their baby continued after birth. This is important, with increasing evidence of the important role fathers’ play, not only in the lives of their partners, but in the health and wellbeing of their children. Fathers who are affectionate, supportive and involved, can contribute positively to their child's cognitive, language and social development.^79^ Children who have more positive relationships with their fathers tend to have fewer behavioral problems at school,^80^ which is strongly linked with higher educational attainment, especially in relation to their levels of literacy.^81-83^

Expectant and new fathers experienced negative feelings and fears relating to not knowing what to expect of their roles, leaving them feeling nervous and unprepared. This uncertainty often resulted in men feeling helpless and excluded, similar to findings reported by Hildingsson and Thomas,^84^ who found new fathers experienced negative feelings about the pregnancy, the upcoming birth and the first weeks of fatherhood with a newborn baby. In the current review, men also expressed fears relating to labor and birth, as well as concerns about their partner's and baby's wellbeing. This is in line with findings of Hanson *et al.*^85^ that, before the birth, fathers often expressed fear for the safety of their partner and the baby, anxiety and fear about observing their partner in pain, feelings of helplessness, lack of knowledge about the birthing process and concerns about risks of interventions such as operative delivery, limited finances and parenting skills. Similarly, in the recent quantitative systematic review by Philpott *et al.*, stress levels in fathers were found to increase in the antenatal period due to negative feelings about the pregnancy, role restrictions related to becoming a father, fear of childbirth and feelings of incompetence about infant care.^23^ High anxiety and depressive symptoms during pregnancy were the most significant predictors of depression in men in the postnatal period,^7^ highlighting the need for better information and support for expectant fathers in the antenatal and postnatal period.

The current review found that, following the birth, men felt excluded from the relationship with their partner as the focus tended to be on the baby, which often left them struggling to find a role.^49,56^ The role restrictions and changes in lifestyle often resulted in stress, which manifested as tiredness, irritability and frustration. These findings are consistent with those in other studies,^23,86^ where feeling pushed out and role restrictions related to becoming a father were contributory factors for paternal stress in the perinatal period. Tiredness and stress, were managed by many men through distraction techniques, such as getting engrossed in work, listening to music or smoking.^50,60^ Denial was another coping mechanism and some fathers felt that they did not have the right to share their concerns or worries as they did not view them as being important.^50,53^ Men's reluctance to discuss their own mental health concerns, due to wanting to protect their partner, and engaging in escape activities such as overwork, sports, sex, gambling or excessive drinking to cope with stress have been reported previously.^87-90^

The lack of social and peer support available for first time fathers was an important finding of this review given the impact on fathers’ mental health and wellbeing. Castle *et al.*,^91^ in a study of 66 first time expectant fathers, reported perceived social support to be a protective factor, with fathers who reported higher levels of perceived social support throughout the pregnancy experiencing lower levels of depression and distress six weeks post-delivery. Poor social support is also associated with antenatal depressive symptoms in fathers.^92,93^ Fathers were unaware of resources that were designed specifically for “dads”, and felt that the services were mainly aimed at women.^50,51^ Many fathers felt excluded by health professionals, who mainly focused on the mother, and often not acknowledged as equal partners in the process. These findings are also consistent with previously identified literature, where fathers have reported feeling marginalized by health professionals during the perinatal period and not having access to appropriate information from the fathers’ perspective on pregnancy, birth, child care, and balancing work and family responsibilities.^34-38^

Evidence from included studies showed that new fathers expressed a need for more guidance around the preparation for fatherhood. This included practical advice around clothing, feeding and routines for the baby, and information around relationship changes with their partner following the birth of the baby, including sexual relationships. Poor couple relationships, feeding difficulties and anxieties relating to the tasks of early fatherhood have previously been associated with poor mental health in fathers in the perinatal period.^4,51,75,94^ Supporting strong couple relationships, engaging with fathers, and supporting the transition to parenthood for first time parents have all been highlighted as priorities for the national Healthy Child Programme in England;^42,95^ however, meeting these needs in practice remains an issue.

Having a variety of support mechanisms in place, including parenting groups that involve other new fathers, resources that are father-friendly and services that are father-inclusive, were perceived to be useful strategies that would support fathers’ mental health and wellbeing. The evidence included in this review, however, did not identify when the optimal time in the perinatal period would be to provide information or support to new fathers in preparation for fatherhood.

Although there were many challenges in becoming a first time father, several positive aspects were identified. Many fathers did not bond with their child straightaway as discussed earlier, but the more time they spent with their child, the more confident they became and reported the experience as extremely rewarding.^50,54-56^ Managing new fathers’ expectations and encouraging them to be involved with their child in the early days would help them to bond and promote better outcomes for the whole family. Fathers who are affectionate, supportive and involved in their child's care and upbringing contribute positively to their child's cognitive, language and social development,^79^ with potential to generate social, academic and economic benefits in the future.^93,94,96^ Conversely, fathers who are disengaged with their children at three months postpartum have been shown as a predicting factor for behavioral problems in children.^97^ Close connections with their children can also lead to positive outcomes for fathers themselves, including satisfaction with family life,^98^ higher levels of satisfaction in mid-life,^99^ and lower likelihood of separation/divorce.^100^ There are other benefits associated with father engagement and health and wellbeing of their partners. Positive father involvement with childcare and household tasks have been associated with lower levels of stress and depression in mothers^101^ and paternal support has been strongly correlated with lower rates of depression in women.^102^

Fathers who recognized and accepted lifestyle changes made better adjustments to the new role of fatherhood^58,61,65^ and couples who were better prepared worked stronger in partnership to address challenges of parenthood.^50,61,65,66^ This shows the importance of adequately preparing couples for the changes parenthood brings and finding ways to enable them to work together and support each other in early weeks and months following the birth. The importance of the quality of the man's relationship with his partner during the antenatal, intrapartum and postnatal period was a key element to the transition to parenthood in the literature review of 32 studies, by Genesoni and Tallandini.^24^

As there are no known previous qualitative systematic reviews on this topic area, the findings of this review have important implications for practice, particularly relating to the way in which care is offered to fathers and families in the perinatal period. It provides evidence from an international perspective of first time fathers’ experiences of new fatherhood and highlights gaps in the current service provision. Healthcare professionals need to be aware of the dilemmas and challenges new fathers face in order to better support their mental health and wellbeing during this crucial period.

### Limitations

It is acknowledged that the included studies lacked homogeneity to a certain extent. Of the 22 included studies, 19 explored the general experiences of expectant or new fathers, while only three focused specifically on the mental health and wellbeing of first time fathers. Furthermore, each study concentrated on different periods of transition to fatherhood. For example, some concentrated on the antenatal period, while some focused on the early weeks following birth and others on the early months. As the meta-aggregative approach used pooled findings based upon thematic or descriptive similarities, these different factors are unlikely to confound the results of the review, but rather add to them by creating a better understanding of new fathers’ experiences throughout the perinatal period.

Although there was variation with regards to age and occupation of first time fathers across the included studies, the lack of ethnic diversity was noted. Of the nine UK studies, participants in six of them were of a White background. The ethnic homogeneity in the UK based studies highlights the need for more research on mental health and wellbeing needs of fathers from other ethnic groups, as these studies do not reflect the ethnic diversity of the UK population. Similarly, participants in the majority of the remaining studies also lacked ethnic diversity,^54,58,60,63,68^ and three studies did not describe the ethnicity of study participants.^47,48,56^

Sample sizes in two included studies should also be considered. The study by Shirani and Henwood^67^ included two first time fathers and the study by Dallos and Nokes^49^ included one. As the main focus of this review was to gain better understanding of first time fathers’ experiences and needs, these studies were considered to be useful, with similarities noted between the findings generated from these studies and the others included in the review.

A limitation of only including first time *resident* fathers means that the mental health and wellbeing needs of non-resident fathers remain unknown. Although this review set out to include non-biological fathers, such as adoptive fathers and stepfathers, the review did not identify any studies on these groups of fathers, highlighting a gap in research around non-biological fathers’ mental health and wellbeing needs during the perinatal period. This review excluded non-English language studies due to resource/time constraints, meaning that cultural, country specific and other insights into the role of first time fathers from a global perspective could not be elicited. Considering such studies in future research could be helpful.

It is acknowledged that as this is a qualitative systematic review, generalizability of results is not possible. However, the studies were carried out across eight different countries and included participants from different age groups, ranging from 18 to 58 years, and various occupational groups from unemployed to higher managerial/professional groups. These qualitative studies provide useful insights in the context in which mental health and wellbeing is experienced by new fathers, as well as rich narrative illustrations from individuals, which provide better understanding of the specific needs from the perspective of first time fathers.

## Conclusions

The aim of this review was to identify first time fathers’ needs and experiences in relation to their mental health and wellbeing during their transition to fatherhood. Three main factors were identified: the formation of the fatherhood identity, the competing challenges of the new fatherhood role and the negative feelings and fears relating associated with it. Role restrictions and changes in lifestyle often resulted in stress, which manifested as tiredness, irritability and frustration. Fathers used denial or escape activities, such as smoking, working longer hours or listening to music, as coping techniques.

More guidance and support around the preparation for fatherhood and consequent relationship changes with their partner were identified as important for first time fathers. Having support mechanisms in place, including parenting groups involving others with similar experiences, father-friendly resources (containing information from a father's perspective) and father-inclusive services were perceived as useful strategies that would support mental health and wellbeing. The main barriers to accessing support included a lack of resources specifically aimed at fathers and lack of health professional engagement with fathers. Many fathers also lacked support from their male work colleagues and peers.

A number of positive aspects were identified. Fathers who were involved with their child and bonded with them over time found their experiences to be rewarding. Those who recognized the need for change in their life and relationships, adjusted better to their new role, especially when they worked together with their partners. Better preparation for fatherhood and support for couple relationships during the transition to parenthood could facilitate better mental health and wellbeing in new fathers, resulting in better experiences of their transition to parenthood.

### Recommendations for practice

The following recommendations are based on the findings from this qualitative systematic review, which represent Level 1 evidence (see Appendix VI for JBI Levels of Evidence Recommendation).^103^ The evidence from meta-syntheses 1, 2, 3, 4, 5 and 7 were rated as “moderate” on the ConQual assessment^1^ and therefore could be used to inform practice. Each recommendation is assigned a grade, either “strong” (Grade A) or “weak” (Grade B) for easy interpretation by clinicians and service users, according to JBI Grades of Recommendation Criteria (Appendix VII).

Health professionals should routinely inform and educate expectant fathers about the changes and challenges they may experience during their transition to fatherhood, and offer information on where they could access appropriate resources and support (Grade A). First time fathers must be better prepared for parenthood, with particular focus on difficulties associated with balancing competing demands. Health professionals play an essential part in ensuring that both parents recognize the importance of their roles within the family and that fathers are enabled to contribute positively to their partner and child's health and wellbeing. Fathers should be routinely encouraged to attend antenatal appointments and, when present, informed about the importance of attachment and how they can bond with their newborn babies, including “skin-to-skin” contact. Fathers should be encouraged to spend time with their babies, holding them as often as possible and engaging in verbal exchanges when changing and feeding them, to help them to develop confidence and skills in parenting. If fathers are adequately prepared, then they are likely to have more realistic expectations about what to expect following the birth, reducing the chances of disappointment in the postnatal period (Grade A). Informing fathers about the importance of their involvement to the child's development and how rewarding this could be to them, could encourage new fathers to develop skills and self-confidence in their parenting (Grade A).

Health professionals should focus on couple relationships, including potential changes to sexual relations, and discuss the importance of this with both parents in the antenatal and postnatal period. This could help couples manage their expectations of parenthood and encourage them to use more positive forms of problem-solving to avoid relationship deterioration during the perinatal period (Grade A). In the UK, health visitors (Specialist Community Public Health Nurses) have been identified to be in a prime position to discuss couple and parenting relationships, which can contribute positively to the mental health and wellbeing of families with new babies.^104,105^ Health professionals should provide new fathers with information about the labor and childbirth process, as well as advice of how they could feel involved with their partner and baby in the early and longer-term postnatal period (Grade A).

Synthesized Finding 4 highlighted the restrictions, frustrations and stresses experienced by first time fathers, how they were manifested and mechanisms that men used to cope with them. Health professionals need to be aware of these, as the signs, symptoms and coping mechanisms in new fathers may be different to those displayed by new mothers. They need to provide fathers with adequate support and resources aimed at reducing stress and improving mental health. Where necessary health professionals should make appropriate referrals for fathers to other professionals (Grade A). Health services need to adopt a father-inclusive model for supporting new parents so that fathers feel acknowledged and adequately supported. There is a need for more father-inclusive resources tailored to address their needs and resonate with their experiences (Grade A).

Based on the ConQual assessment, Synthesized Finding 6 was rated as “low”, indicating that the finding should be considered with caution. Although the recommendations for practice are unlikely to have negative impacts and could enhance and better prepare fathers for their transition to fatherhood, the evidence supporting its use was considered ”weak”, therefore this synthesized finding was given a JBI Grade B level of recommendation. Expectant and new fathers should be offered practical advice, information and guidance around caring for their new baby, to include bathing, feeding and sleep routines for the baby. A variety of sources of support should be offered, including face-to-face contacts, online resources and DVDs. Resources need to be evidence-based and credible. This will allow men to choose type of support that suits them (Grade B). Health professionals working with first time fathers should routinely inform parents about their ability to assess perinatal mental health and their role in supporting paternal mental health and wellbeing during this period. However as the ConQual rating for this finding is “low”, it is not known whether this would be acceptable or welcomed by new fathers.

### Recommendations for research

The fathers in the primary studies included in this review lacked ethnic diversity. Considering the cultural diversity of today's society in most high-income countries, further research including first time fathers from different ethnic and cultural backgrounds would provide a much broader understanding of fathers’ mental health and wellbeing needs during their transition to fatherhood.

As this review only included first time resident fathers, the mental health and wellbeing needs and experiences of non-resident and/or subsequent fathers remain unknown, which is another area that could be considered for future research. This review did not identify any studies on non-biological fathers, such as adoptive fathers or stepfathers, highlighting a gap in research around these groups of fathers’ mental health and wellbeing needs during the perinatal period. Studies not published in English were not included due to time/resource constraints; however, including these in any future systematic reviews may provide further useful information or insight from certain countries and cultures.

The findings relating to *what new fathers want* (Synthesized Finding 6) needs further exploration as the rating of confidence (ConQual) was “low”, limiting its ability to inform practice. To better support first time fathers’ mental health and wellbeing during their transition to fatherhood, it is important to establish what support new fathers want and what interventions would be acceptable to them. While this review identified that first time fathers would like support through a variety of sources, the evidence for this recommendation was considered to be ”weak”, suggesting the need for further research into the type of support that fathers may want, how it is provided and by whom, and when the optimal time in the perinatal period would be to provide this. Another aspect that remains unclear is around the routine mental health enquiry or screening for new fathers by health professionals. It would be helpful to carry out further qualitative research in this area to ascertain men's perceptions and receptiveness of this.

## Acknowledgments

This review is being undertaken as part of a Clinical Doctoral Fellowship for SB, funded by the UK National Institute for Healthcare Research.

## Funding

SB is funded by a National Institute for Health Research (Clinical Doctoral Fellowship, ICA-CDRF-2015-01-031). This paper presents independent research funded by the National Institute for Health Research (NIHR). The views expressed are those of the author and not necessarily those of the National Health Service, the NIHR or the Department of Health and Social Care. DB and JS are supported by NIHR Collaboration for Leadership in Applied Health Research and Care South London.

## Appendix ll: Search strategy for MEDLINE (17/03/17)

1. Father^∗^.mp. [mp = title, abstract, original title, name of substance word, subject heading word, keyword heading word, protocol supplementary concept word, rare disease supplementary concept word, unique identifier]
2. Men^∗^.mp. [mp = title, abstract, original title, name of substance word, subject heading word, keyword heading word, protocol supplementary concept word, rare disease supplementary concept word, unique identifier]
3. Paternal.mp. [mp = title, abstract, original title, name of substance word, subject heading word, keyword heading word, protocol supplementary concept word, rare disease supplementary concept word, unique identifier]
4. Dad^∗^.mp. [mp = title, abstract, original title, name of substance word, subject heading word, keyword heading word, protocol supplementary concept word, rare disease supplementary concept word, unique identifier]
5. postnatal.mp. [mp = title, abstract, original title, name of substance word, subject heading word, keyword heading word, protocol supplementary concept word, rare disease supplementary concept word, unique identifier]
6. perinatal.mp. [mp = title, abstract, original title, name of substance word, subject heading word, keyword heading word, protocol supplementary concept word, rare disease supplementary concept word, unique identifier]
7. prenatal.mp. [mp = title, abstract, original title, name of substance word, subject heading word, keyword heading word, protocol supplementary concept word, rare disease supplementary concept word, unique identifier]
8. antenatal.mp. [mp = title, abstract, original title, name of substance word, subject heading word, keyword heading word, protocol supplementary concept word, rare disease supplementary concept word, unique identifier]
9. antepartum.mp. [mp = title, abstract, original title, name of substance word, subject heading word, keyword heading word, protocol supplementary concept word, rare disease supplementary concept word, unique identifier]
10. Mental Health/
11. Stress, Psychological/ or Mental Disorders/ or Mental Health/ or Anxiety/ or Depression/
12. Mental Health/ or “Quality of Life”/
13. Emotions/ or Expressed Emotion/ or Stress, Psychological/
14. Stress Disorders, Traumatic/ or Stress Disorders, Post-Traumatic/ or Stress, Psychological/ or Stress, Physiological/
15. Depression, Postpartum/
16. Anxiety/ or Depression/ or Depressive Disorder/ or Depression, Postpartum/ or Risk Factors/
17. Anxiety/
18. distress.mp.
19. 1 or 2 or 3 or 4
20. 5 or 6 or 7 or 8 or 9
21. 10 or 11 or 12 or 13 or 14 or 15 or 16 or 17 or 18
22. 19 and 20 and 21
23. limit 22 to english language
24. limit 23 to yr = “1960–Current”

## Appendix IV: Study findings with illustrations and assigned credibility level

**Finding 1:** Renegotiating paid employment and household work or childcare work [U].

“I hope I’m around in those times when he is learning to play. There is a couple of hours each day when he wants to play and try and talk and stuff. Because I’m at work I hope I don’t miss out on that too much. I don’t want to come home all the time and [find] him asleep”^48^^(p.1015)^

**Finding 2:** Expectations and symbolic meaning of fatherhood [U].

“I thought as a father there would be a bond there straight away with the child. I thought it would just come naturally. I thought because he was mine I was going to be immediately attracted to this child and love would just come naturally. I was surprised I wasn’t overcome with feelings for him straight away”.^48^^(p.1017)^

**Finding 3:** Changing relationship with partner [U].

“The first week was great, then after that things started to get worse. I never thought that Jenny and I would have fought so much”^48^^(p.1018)^

**Finding 4:** New fathers wish to father differently from their own fathers [U].

“My father was more removed, I’m much more hands on, my father sat around and did little, my experience is very different, I change nappies, make milk and get up in the middle of the night.”^49^^(p.74)^

“In our case the baby's mother is going back to work full time and I’ve decided to finish work to be with the baby. A lot of fathers spend so much time at work. Although we’ve got some financial worries I’d prefer this than not being with my child. I’ve always wanted to be with my child. I want to be around for my child in a way my father wasn’t for me.”^49^^(p.77)^

**Finding 5:** What it means to be ‘male’ [U].

“My father wasn’t around much when I was growing up, he was a coal miner and had lots of bravado and machismo. Bonding with your child is important I think, you get more in touch with what it really means to be male.”^49^^(p.74)^

**Finding 6:** Lack of guidance and obstacles for achieving new fatherhood [U].

“I felt shit scared. Having a new baby is a worrying time and I feel I lack a bit of confidence… fathers are invisible to some of these facilities– facilities don’t take fathers into account.”^49^^(p.75)^

**Finding 7:** Determination and sustained effort required to challenge the constructions of fatherhood [U].

“I had to take the initiative with early childhood services, I had to push to get involved–men have to take more initiative in services, but if they push they get what they want.”^49^^(p.76)^

**Finding 8:** Challenges of combining new fatherhood and traditional Narratives [U].

“I’m having huge difficulty performing across all areas of my life since the baby. I have less sleep, less sexual activity and there's more strain generally. I’m stressed out and drinking too much but the baby has given me an enormous sense of there's more to life than working and having a relationship with one person. Fatherhood amplifies the meaning of life. I’ve organised my business to take days off during the week to be with the baby but I make it up at night.”^49^^(p.79)^

**Finding 9:** Worry about being able to manage being both a good provider and a ‘hands on’ father [U].

“I have to fight to stop work taking over my life. I’m pretty exhausted but I want to be ‘hands on’ with the baby. I like teaching him, naming, climbing and I’m looking forward to teaching him footy and how ants work. I enjoy time with him but he wears me out with the toys, I feel glad to get away but I’m glad to come back. I’m much more stressed than before and worried because my work performance has dropped.”^49^^(p.81)^

**Finding 10:** Engaging with Traditional Fatherhood [U].

**“**It's difficult with building up a new business (dentistry), but we’re managing. I’m feeling pretty depressed and I get worried about the mortgage. We can’t have everything at present. I’ve taken up smoking in the hope it will make me less stressed.**”**^49^^(p.82)^

**Finding 11:** Not Engaging with Fatherhood [U].

One father says of himself: ‘Drinking and drugs are my biggest problems.’

His partner and mother of their child formulated the problem this way:

“He's having problems adjusting to being a parent. He avoids me and the baby. I’m lonely with him in the next room drinking. He doesn’t want to be here–it's the influence of his friends. He doesn’t want to realise there's a third person in our lives.”^49^^(p.84)^

**Finding 12:** Excitement thwarted by partner's reticence [C]

“I could see in the (pregnancy) book there was a lady there and you could see her shape changing, her body shape changing, and I wanted to see that with Esme, but she wasn’t into that at all. She, she was, I don’t really know, didn’t want me to take a picture (of her shape changing). I felt upset, I felt a little bit “oh, come on! Come on!” And I wanted to… but I tried… I did try to, well… I kept it back in a way, because she kept telling me to keep it.… “I don’t want it to be pushed, she’d say, I don’t want… I’m doing this!” Um… I felt a little bit out of it too.”^50^^(p.153)^

**Finding 13:** Relationship deterioration [U]

“Our relationship between the two of us has deteriorated quite drastically now. We are actually going to see Relate.… We go to Relate, we’ve been to Relate twice because Esme suggested we’d better go to Relate because we were, really we were, our relationship is not touching, not talking, nothing, nothing”.^50^^(p.153)^

**Finding 14:** The focus shifting from us to him [U]

“You run around after (the baby) whereas I felt that I… I felt that he could… he would join in with my life or our life. He would be… I always felt that I was in this relationship with the two of us and he would be the addition to it. Whereas now I feel that he is, he is the life and we are running around after him”.^50^^(p.154)^

**Finding 15:** Feeling left/pushed out [U]

“And I felt really out of the whole thing.… I wasn’t involved in that (the pregnancy)… I couldn’t be because it wasn’t in me… and all I could do was be there for her”.^50^^(p.155)^

**Finding 16:** Wanting to cherry pick the best bits from own childhood [C]

“He wanted to parent Alfie in an optimum way and be discerning by “cherry picking” the best bits from his own experience of being fathered”.^50^^(p.155)^

**Finding 17:** Wanting to bring baby up in best way [C]

“One thing I kept saying to Esme is, “I am an engineer, I can do things precisely. I could build this, this table precisely. I could screw it, but the screw has to go in a particular place and the top goes on the top and the legs go in the right directions so they are a precise science.” So I wanted to get this…the baby, I tried to organize this baby in precise ways. Getting a baby monitor, the cot goes there, nappies can go there, that, that, that's how I (pause) treated the whole childhood thing, the baby, um, yeh”.^50^^(p.157)^

**Finding 18:** Wanting to get things right [U]

“I am also worried of not getting it right. Uh… do I let him play on the floor with the baby gym with all the things hanging all over the top; he's interested in that. But do I, do I leave him or not? Do… er… is that not interacting with him enough? But then, if I put him in the cot in his springy seat thing, but what am I supposed to say to him? Am I supposed just to play with him? Cuddle him? Am I supposed to? And… and I don’t naturally sort of feel, I don’t know what to do”.^50^^(p.156)^

**Finding 19:** Worries about being a good enough dad [U]

“One minute he's over there being fed, then he's being winded, then he's on the floor in the baby gym, then he's up on his spring seat over there, and then he's upstairs in his cot, then he's back.… I don’t know, and he doesn’t know, you know, bouncing in the doorway, he doesn’t quite know, I don’t think he quite knows what he's up to, up to, because I’m worried that I’m not going to be good enough, I’m not being good enough”.^50^^(p.156)^

**Finding 20:** Struggling to find a role [U]

“Ah, and I’ve been struggling in a way to try and find what… what is my role with this child. Um, is it to do as (Esme) does, i.e., feed him, wind him, change his nappies, bath him, clothe him? Do all those things. Everything”.^50^^(p.157)^

**Finding 21:** Life's restrictions on becoming a parent [U]

“One of the feelings I have been getting is of… I can’t do all the things I want to do. I found it very frustrating.… I’ve been on leave for quite a lot recently.… I find it very frustrating when I can’t, I can’t get to go and do something I want to do like… like the washing… something simple like that”.^50^^(p.158)^

“Um… I didn’t quite understand, I don’t think I quite understood how full on babies are. Er… they’re 100% and more. They take over your life and there's no… you don’t have a life in effect really”.^50^^(p.158)^

**Finding 22:** Apprehension about criticism [C]

“I enjoy that (taking Alfie to the health visitor). I enjoy getting involved with it, but… Esme… tends to take over… she seems to feel that she's the mother… that I can’t do it properly”.^50^^(p.157)^

**Finding 23:** What is expected of men is different to how I feel! [C]

“But I suppose as a man I think… it's always been a perception that we’re supposed to able to handle it… we’re supposed to be able to get on with it. We’re not supposed to get upset about things. Esme only ever asks me what I am thinking… “Is everything alright?”… if I’m upset and she can see that I am physically upset… I’m… I’m crying. If I’m not crying she won’t ask. I don’t think she expects me to be upset or possibly even be…want to talk about something.”^50^^(p.158)^

**Finding 24:** Male friends at work unable to offer support [C]

“I mentioned (at work) we were going to Relate and… uh… there tends to be a, “oh,” and that's it really. You don’t have much of a heart to heart with blokes. Um… but it's been nice in a way just to say something.”^50^^(p.158)^

**Finding 25:** Going to work/wanting to parent [U]

“Yesterday, I didn’t see him very much because there was… when I went to work he was in bed and when I came back he was in bed, and I didn’t see him at all… probably it was an hour yesterday, which I felt wasn’t sufficient connection. So when I saw him today, I felt “Look, it's Dad! Please, I’m Dad!” You know, “Please recognize me! And don’t forget me!”^50^^(p.158)^

**Finding 26:** Legitimacy of paternal stress and entitlement to health professionals’ support: Articulating and attributing stress [U]

“I think for me it's just – the never having any time to relax, it's just not possible. I’ve got a stressful job then I come home and I tend to get … the tired, stressed baby … I think the stress for me is just the non-stop-ness of it.^51^^(p.5)^

**Finding 27:** Legitimacy of paternal stress and entitlement to health professionals’ support: Symptoms and manifestation [U]

“I tend to do the typical man thing of hiding it until I can do so no longer. … I’m not the sort to wail and shout and whatever. … I probably just get grumpy and a bit snappy about stuff. That's pretty much it really”.^51^^(p.5)^

“Yes, I could feel myself withdraw, so I wouldn’t communicate as much and I would get snappy when sometimes I wouldn’t do. It was something that if I was already close to it, it would be the minutest of things that sometimes would just make me lose it, not lose it, but kind of just [pause].^51^^(p.6)^

**Finding 28:** Legitimacy of paternal stress and entitlement to health professionals’ support: Entitlement to health professionals’ support [U]

“I think at the birth I felt a bit more like a spare part, but then again I mean they were really good with [partner], I just felt in the way sort of thing”.^51^^(p.6)^

“[The midwife]'s interested in [partner] and knowing that I was supporting her, but not so much as me, which, they can’t involve everyone, or take a responsibility for everyone … I very much felt like it's certainly not about me, this. But at the same time, I do very much appreciate the limited resources. They can’t be responsible for everyone. The pregnant woman is the priority, isn’t she”.^51^^(p.6)^

**Finding 29:** Protecting the partnership [U]

“I struggled at times because whilst I could see of the physical effects on [partner], I couldn’t understand the emotional and mental effects it was having on her, so I struggled with that, and I probably did become a bit more snappy, definitely low mood at times and struggling to sort of sleep properly, and you have a lot to think about as well so you’re trying to do everything, trying to make sure that we’re ready but also ready with the house and you’ve got so much to sort of think about”.^51^^(p.7)^

**Finding 30:** Navigating fatherhood: Feeling prepared and (changing) expectations [C]

Some men reflected on the importance of changing their expectations, acknowledging that some of their stress reflected an unrealistic standard that they and their partners had set for themselves:

“Even though it wasn’t by the book, but it made our lives a lot easier and that I think helped as well, not listening to what everyone told us”.^51^^(p.8)^

**Finding 31:** Navigating fatherhood: Managing stress through distraction, denial and release [U]

“I’m probably the sort of bloke who actually just says, ‘oh I’m quite forgetful, so I can forget I’ve had the worst night ever’. I just try and forget it. So that's probably my coping mechanism. It's just, trying to forget it and I generally do. And then, I guess, I’ve found in some ways, work quite helpful in that respect, because you can have a crazy night where you have no idea what's going on with [son's name], but I can go to work and I feel fine. I’m in control here, I know what to do. There's people who I can actually communicate with, they’ll do what I ask them to do and vice versa. So I’m probably not the best example, the best person to ask, because I think I just choose to ignore. I’m probably more of an ignorer, which isn’t probably that helpful for [partner].”^51^^(p.8)^

**Finding 32:** Navigating fatherhood: Strength through fatherhood as rewarding [U]

“The sleepless nights do take their toll on you, but I don’t know if it's just the way that I think … but I tend to look at the bigger picture. I just think I’m happy because she's healthy, she's smiling… So I think, well, I must be doing something half right for her to be trotting around as she does, and she's happy with me”.^51^^(p.9)^

**Finding 33:** Diversity of men's support networks: Pre-existing networks – friends, family and the wider community [C]

“[At work] I can cover an awful lot of different things with them… And in a lot of cases, it is bloke banter. You wouldn’t think that it [but] you’re in the middle of an engineering workshop surrounded by blokes, and we probably spend half the day talking about babies and kids and that sort of thing. But I feel more comfortable with it, because I know that there's guys there that have had similar experiences or they know what it's like. They know how I’m feeling if I say, oh, we’ve had a rough night … Some people have had worse experiences, so you think, what we’re going through is normal.”^51^^(p.10)^

**Finding 34:** Diversity of men's support networks: ‘Formal’ peer support and opportunities to meet other fathers [C]

“I think in some ways it would be helpful before and after to make sure that dads are prepared and that they’re coping and maybe even if it was just away from the mums for some people maybe, because I think some dads might find it a bit embarrassing to sort of say I don’t know what I’m doing.”^51^^(p.10)^

**Finding 35:** Diversity of men's support networks: Lack of information resources tailored to men [C]

“I wouldn’t know if there is anything, the equivalent for dads, I’ve not really set out to look that specifically, I’ve just come at it more as being a parent … I absolutely would [feel comfortable using netmums] more than happy to look for help, advice, and other people's experience anywhere really”.^51^^(p.10)^

**Finding 36:** Information [C]

The Haynes Baby Manual (Banks 2003) was the only publication mentioned that was aimed at men:

“Oh, I’ve got my Haynes manual…It's the Haynes manual for babies, a guy at work whose wife had a baby recommended it to us”.^52^^(p.629)^

“Apart from this, frustration was expressed at the lack of information intended specifically for new fathers”.^52^^(p.629)^

**Finding 37:** Involvement in healthcare provision [C]

“The classes are a great help, but if you’re not involved in it, you’re sort of put to the back of the class, so to speak.^52^^(p.629)^

**Finding 38:** Support [U]

I would have, yeah, really struggled to have anyone to go to yeah, because…the care is, it is very much geared towards the women.^52^^(p.629)^

**Finding 39:** Preparation for fatherhood [C]

“I would look now to wanting more information about what to do when I’ve actually got it…even little things like what clothing, when you put it to bed, getting into a routine, even the basics, really”.^52^^(p.630)^

**Finding 40:** The birth [C]

“Just knowing the facts around the caesarean. It wasn’t discussed, and I wasn’t prepared for it…I wish I could’ve helped, know what to expect…that really upset me for a while”.^52^^(p.630)^

**Finding 41:** Parents’ relationships [C]

“You are both tired, niggling at each other, and it was probably slightly worse from what we thought. I mean, if the awareness could have been made a lot more, because no one ever really spoke to us about that other side…the relationship with us and the baby. We sort of sat down and we tried about two or three different ways and thought about this”.^52^^(p.631)^

**Finding 42:** Fatherhood [C]

Overwhelming feelings for the baby were commonly talked about: amazement, love and a sense of great responsibility, surprise and confusion in the first few weeks. Having the baby was a completely life-changing event:

“It was all such a shock, suddenly. You’re prepared but, you thought you’d prepared for it but…^52^^(p.631)^

**Finding 43:** Self and Other as Individual: Coming to terms with the physical and emotional changes during the postpartum period [U]

“The first night after the birth, it was time I lie down, I was so tired. I wasn’t worried, just exhausted,”^53^^(p.331)^

“Taking care of my wife, and then the baby, I became so tired.”^53^^(p.331)^

**Finding 44:** Self and other as a parent: coping with parental demands [U]

“Breastfeeding was what I found most difficult. I didn’t know how to help, I felt useless.”^53^^(p.333)^

“At times, my wife had difficulty breastfeeding, it made me so anxious. I just wanted the baby to drink well,”^53^^(p.333)^

**Finding 45:** Self and Other as a Couple: Maintaining Conjugal Functioning [C]

“During the hospital stay, we would take time to be together just the two of us, but the feeling of closeness was different, as if I could only see my baby's mother in her and not my spouse. It was more difficult between us, ore tense, the whole situation was more tense than easy”.^53^^(p.333)^

**Finding 46:** self and other interacting with the environment: coming to terms with environmental demands [C]

“I was filling out forms on breastfeeding, nobody had explained them, yet I made some sense of them. Despite that, they kept changing the time she should breastfeed, and nobody explained why”.^53^^(p.333)^

“It was one feeding after another; I was under the impression of having no respite. I knew it would be like that, but I still found it difficult”.^53^^(p.333)^

**Finding 47:** self and other interacting with nurses: exchanging information with nurses [U]

“It was important to me that all the involvement I had had during pregnancy, childbirth and now, after, be recognized by someone else than my spouse. I wanted others to be able to recognize my involvement, by simply talking to me, by including me in conversations. I wasn’t excluded by nurses, they didn’t ask me to leave the room, but it was a nonverbal exclusion, by the way their body was… they never asked me how I felt as a dad”.^53^^(p.334)^

**Finding 48:** The perceived positive relationship between being male and the ability to father children [U]

“Over the moon…I suppose it's like a man thing. It's like you feel more of a man in a way. I know it sounds a bit weird but you feel more a man…You feel everything's working and you’re alright. So I was over the moon, overjoyed”.^54^^(p.1023)^

**Finding 49:** Maintaining health to meet the needs of forthcoming dependents [C]

“I’m not one for boozing all the time …. But work has to come first now. I have another person to think about now… There's no two ways about it. You have to change”.^54^^(p.1023)^

**Finding 50:** Childbirth perceived as a shared experience and being there [C]

“I’ll be there…doing what she wants when she needs it. I’ll block me ears when the foul language comes out”.^54^^(p.1024)^

“I’ve seen all the movies and all the things on the TV…I’m going to be there to hold her hand…I’m sure I’ll get told off!”^54^^(p.1024)^

**Finding 51:** Lack of knowledge about childbirth [U]

“It could be a bit more directed towards fathers. As regards information … There could be a bit more for fathers. There could be a little booklet telling you all the information you need”^54^^(p.1024–1025)^

“I suppose a bit nervous and frightened. Because I don’t know what to expect. Well I do and I don’t. But it's the first time so I don’t know really what to expect until it actually happens”.^54^^(p.1025)^

**Finding 52:** Aspects of the labour and birth [U]

“I want to be up the head end…I don’t want to see any of that end at all because I don’t like it, at all…That's the only thing I’m worried about”.^54^^(p.1025)^

“First and foremost I hope I don’t pass out. Because I don’t like needles and all that sort of stuff…It just sends me a bit funny…I’m hoping I won’t pass out anyway. But you never know”.^54^^(p.1025)^

**Finding 53:** Disclosing personal difficulties [U]

“I have concerns and worries about things … But I don’t have the right to share those because she's going through all this. She's going to have all this pain and everything else… My little worries are not really that important in the light of things”.^54^^(p.1026)^

**Finding 54:** Social support [U]

“No, I’m not a person for sharing my problems with other people”.^54^^(p.1026)^

“They just take the micky really … keep telling me my life as I know it is over [laughs]”.^54^^(p.1026)^

“I tend to find that women stick together and they talk about girlie things and babies and stuff. And they tend to keep it to themselves”^54^^(p.1026)^

**Finding 55:** ‘Being there’: men's experiences of the labour and birth – presence during labour [C]

“I’m not the greatest person with needles and blood… But I was fine. I was more focused on [partner] and how she was feeling than thinking about what I was feeling”.^54^^(p.1028)^

“They were coming and checking her every couple of hours and every time they asked me to leave…They’d say ‘Do you mind going out I’m going to check her’…At the time you don’t think. You do what you’re told”.^54^^(p.1029)^

**Finding 56:** ‘Being there’: men's experiences of the labour and birth – Caesarean [U]

“I just wanted it to be over with”.^54^^(p.1029)^

“You’re worried, you’re anxious, you’re scared…You don’t know what's going on. You want the end product like but obviously you don’t want the end product to…for anything to happen…I just wanted them to make the decision and get in there”.^54^^(p.1029)^

**Finding 57:** ‘Being there’: men's experiences of the labour and birth – Healthcare professional [C]

“When you go in [the labour room] there is a bed and a chair…Your expectation is that's your chair and you don’t move. It's all lined up like that…There is a chair next to every bed at the head…so like you know your place when you go in…But that wasn’t the case …Every time they got me involved…when they took me through it…that was an extra for me…[Later in the interview] I didn’t think they would get me involved as they did… [But] I don’t think I would have walked away thinking, ‘Oh I wasn’t involved’. Because the emotion of seeing your daughter born…being there to see it would cancel that out…I am grateful that they did get me involved, but I don’t think it would have made the day any worse if they didn’t”.^54^^(p.1029)^

**Finding 58:** Feeling of unreality [C]

“….from the beginning it was very unreal. Accordingly I walked about, was happy, told everybody and became ‘high’, I’m daddy just like you are! But,…..then it isn’t so obvious when it isn’t visible so it's not there….”^55^^(p.98–99)^

**Finding 59:** Feeling of insufficiency and inadequacy [C]

“….but, then you can’t get away from these small nervous elements which come the whole time, I mean the moments of insecurity in the matter about exactly how one should deal with it, partly my woman's fear, on different occasions, about the pregnancy itself, but also about what is coming. How one should practically manage everything that will come afterwards and will be for the rest of my life”.^55^^(p.100)^

“….then I got such a suffocating feeling about becoming a father. I got it continuously. I got a feeling that I would always have a bad conscience. If I’m doing something just for myself…..This is a scary thought….. I can’t live that way, I can’t give up MY life”.^55^^(p.100)^

**Finding 60:** Feeling of exclusion [U]

“…she said hallo to my wife and turned her back on me so I had to push myself forward, in front of her, so that I could shake hands with her as well. For the first five minutes she only looked at my wife and spoke to her alone ‘What do you think?’ (Edward) (Translators note–the singular form of ‘you’ was used in the Swedish)^55^^(p.100)^

**Finding 61:** Feeling of reality [C]

“I think it was enormously moving, I started to cry,… so it was so, soy, a human being is living here inside? It was, still only such a little thing….It was the first ultrasound, I felt enormously taken….Then it was in the sixth, seventh, eight month, then everything was wonderful. It's obvious to me that she is growing every day. Everything works when we go to listen to the heartbeats, to ultrasound and so on. It's like I am able to share something which is real”^55^^(p.100)^

**Finding 62:** Feeling of social changes [U]

“I have noticed that my friends and I have drifted so incredibly far apart from one another during these nine or eight months, yes it actually happens….. it's tedious,..but they will come back when they are in the same situation…hopefully”.^55^^(p.101)^

**Finding 63:** Feeling of responsibility [U]

“….there was something in the breadwinner factor that made me feel that I should change my priorities. It happens even before the baby is born. We are building our ‘nest’ and making more rational decisions then before”.^55^^(p.101)^

**Finding 64:** Feeling of development [U]

“I feel, that I’m growing, as a human being. Yes, it's what I’m doing, absolutely. And even as a man. That it's undeniably one kind of confirmation”.^55^^(p.102)^

**Finding 65:** Expectations [U]

“It's like hitting a brick wall It's like, when they put something up, you know it's going to be there but until you actually get there you don’t know what to expect”.^56^^(p.296)^

**Finding 66:** Reality[C]

“But now J [wife] will take the opportunity to take a bath that she doesn’t get to do during the day. Everything has to be done in shifts now. Before we could sit down and be together. You spend so much time focusing on the baby you forget about each other”.^56^^(p.296)^

**Finding 67:** Transition to mastery [C]

“I noticed for the first 2 weeks he was home I was still living my same lifestyle. I go out once in a while and I’d just leave them home, and I think right then is when don’t to myself, I don’t feel any part of this, and the important thing I think for a father to do is to get involved. The more you get involved the more rewarding it becomes like when you get his first smile or his first laugh.”^56^^(p.296)^

**Finding 68:** Expanded role of good fathers [C]

“I think being there for all their first major things is important, i.e. when they’re at school, when they go to do a nativity play, going to the nativity play, not saying no I’m too busy at work or, you know, someone will video it for me, or whatever”^57^^(p.343)^

“You just have to put yourself second, your child comes first and yourself comes second”^86^^(p.343)^

“A parent who is prepared to put work second and family first, you know, the father who's prepared to do that, I think that's a good father”^86^^(p.343)^

“It is about… being understanding as to what his needs are really, and taking pleasure from watching him develop really… to sort of take him from being very unhappy and crying to get him laughing, getting him engaged in something by I don’t know… making up silly rhymes about him or something taking Daniel from being unhappy to making him happy without sort of forcing him to do anything”^86^^(p.343)^

**Finding 69:** The caring father might emerge as, in fact, the bigger bloke [C]

“So I suppose if blokes are being macho whilst they have a child he is trying to prove his masculinity, I think in my mind they can prove it by being a bloody good father and true with their emotions.… I think it is a brilliant experience. I think a lot of men give themselves bad press on it, it is not big and macho to go down the pub and ignore your child. And it is not big and macho not want to change a nappy, you have got to get involved, and you have got to get involved now or you will lose it”.^57^^(p.344)^

**Finding 70:** The pleasures, benefits and rewards of bonding with their child [U]

“I think the nicest bit is just spending time sitting around on the bed and just playing with him, and just talking to him and being talked back at, and changing his nappy when that happens as well and, you know, time looking at him and him looking at me really is the bit that I’m really enjoying”^57^^(p.344)^

“I do like feeding him and I do enjoy that. And after the feed he just snuggles up to you and he gets his head right into your neck, and that is lovely. And I do and if I fall asleep with him it is fantastic lying on your chest, and I do enjoy that”^57^^(p.344–345)^

**Finding 71:** Tensions and difficulties: Cash and/or care? [C]

“I feel as though my work, because my family's number one my work's got to be number one at the moment and it's that, it's that absolutely what seems to be an irreconcilable tension between the fact that you work, you are working for your family and you’re trying to build a career. Because you know you want to spend, you’re trying to build a career because you want the time and the quality time to spend at home. And you’re building a career and as a result you’re not getting that quality time to spend at home. So you’re wanting both and if you don’t have one you haven’t got the other half, you know its um its really frustrating”^57^^(p.346)^

**Finding 72:** Whose needs? Whose values? Selflessness and autonomy in dialogue [C]

“I don’t know really where it comes from, probably it is to do with the responsibility thing really. That you know she is my offspring and I probably ought to spend more time with her… Um I don’t really know, it just seems to be as things probably ought to be ideally… but I don’t feel very keen on, I know it's coming but I don’t feel very keen to have to, er, erm, sacrifice my time basically, because I, I spend most of my time, erm, well quite a lot of my time, sort of renovating houses and I, fiddle with practical things and, er, to be honest, babies don’t interest me greatly”^57^^(p.347)^

**Finding 73:** Helping out’ or ‘full involvement’? Fairness, equity and decision making [U]

“I think there's an issue with me partner that, and we have touched on it, that I don’t want all decisions made by her, I want it to be discussed, I want it to be fair. But obviously then what I’ve also got to appreciate, that if I’m a hundred miles away, as I am quite regularly, and although we can discuss things on the phone, she might have to make a decision quicker than that, in which case she makes the decision, doesn’t she? So I’d be worried about not being involved in some decision-making”.^57^^(p.349)^

**Finding 74:** On the inside, looking in [U]

“She's rubbing him every day and she's got that contact and she feels him kicking all the time. So yeah, I’m removed from that, aren’t I? And I think, as I said to feel that, him kicking his dad, kind of yeah, definitely gave me that physical contact that [wife] is probably quite used to. But it is quite distanced because you’re not … you’re not developing the baby are you?”^58^^(p.1008)^

**Finding 75:** Present, but not participating [U]

“[W]e’re expected to do a lot more these days, we’re expected to be a lot more involved … but when we are involved we’re still a bit on the outskirts from what I’ve seen … I don’t know if it's because a lot of women don’t take their partners with them, I’m not sure how other people work. It's sort of maybe they’re not always used to having a man there as well, but it would’ve been nice to be acknowledged a little bit more, just so you feel a bit more part of it more than anything. Because you feel a bit awkward sometimes just stood there like ‘Should I wait outside?’ “^58^^(p.1009)^

“I felt as if I shouldn’t be looking kinda thing, you know, ‘coz when you grow up and people pull the curtain across, it means you shouldn’t be looking in there doesn’t it or it's like a private area. Even though it's my wife, I’m kinda thinking, I think I might have even backed into the err, behind the curtain when I went to record off the – off the machine on the wall. But it made me feel quite uneasy to be honest”.^58^^(p.1009)^

**Finding 76:** Deference and support: a moral response [C]

“I didn’t know how to help her. And I think that's frustrating. Really frustrating where you can’t, you can’t do anything. And, you know, you try and do everything you possibly can, you know, make sure she's eating the right things, used to sit there reading the Internet trying to, you know, what can make her better, and speak to as many people, mums and stuff, and see, you know, what can – but she was going – I think it was really hard for her, she had the worst part of it”.^58^^(p.1011)^

**Finding 77:** Feeling Like a Father [C]

“I don’t feel myself as a father, or how should I put it… I don’t feel it consciously. It was not like going up stairs and at a certain point, “I’m a father from today!” Such a feeling didn’t come to me. It was more like going up a slope”.^59^^(p.162)^

**Finding 78:** Realizing Oneself as a Husband [C]

“I know I’m a father, but I think the most important person for me is my wife.… I don’t think my daughter wins her in this sense.… I became a father, but the number one should be my wife. I like to keep this feeling in my mind. And I want my daughter to see me in this way”.^59^^(p.162)^

**Finding 79:** Finding the wife's pregnancy and delivery for the first time to be an impressive experience [C]

“I think I have done almost everything that should be done. Many of them were first experiences for me. For example, I visited a shinto shrine for praying an easy delivery, which I think is unique to Japan, and I also bought an obstetrical binder and child-related products”.^59^^(p.162–163)^

**Finding 80:** Sharing time and space with one's child [C]

“Nine months ago, it was like she suddenly started to cry. It was like an alien, or maybe a strange creature. But she started to show some gestures, or smiling, or show various expressions. I thought it was a change”.^59^^(p.163)^

**Finding 81:** Being aware of a change and trying to adjust to a new life [U]

“Talking about meals, if at restaurants, I’m afraid that my daughter will cry to bother people, so I come to think of eating at home. I think our eating style has changed. But for me, it's not something inconvenient, unpleasant, nor restricted. Rather, I am enjoying the time”.^59^^(p.163)^

**Finding 82:** Being aware of the difference between oneself and one's wife [U]

“Not only when it comes to breastfeeding, but also when it comes to sleeping, my daughter falls asleep more easily. Maybe she is more reliable on my wife than on me.… When she is crying, feeling uncomfortable, or feeling sleepy, she jumps into her mother. So when I see such a situation, I feel like, “Why don’t you come to me?”^59^^(p.163)^

**Finding 83:** Grappling with the reality of the pregnancy and child [C]

“It's more in my head, I know she's pregnant, but there's nothing to feel yet (9 weeks gestation).”^60^^(p.13)^

“My experience is that there is a child that is supposedly happening. All we have is this test that is pretty reliable….Although I was ecstatic when she showed me the test, I don’t see anything happening yet. It's all from her, and I feel like I probably won’t get on board…until I hear it from the doctor. “(7 weeks gestation)^60^^(p.14)^

**Finding 84:** Struggling for recognition as a parent from mate, co-workers, friends, family, baby, and society [U]

“It's always in reference to how [my wife] is doing, and I feel like I have resigned myself more to just responding to what they are asking and that is to say how [she] is doing as opposed to me and how I am doing….I really tried to initially go out…and open myself up and really share…but, so much of the response is, ‘You’ve just got to stick it out. This is her time.’ There is no validation of the feelings. There is no recognition. I don’t feel like I should deny my feelings and deny what's going on for me. The message is clear…’You need to focus on her.’ I just haven’t found anybody that is real understanding, like ‘What is the experience like for you?’ (37 weeks gestation)^60^^(p.14)^

**Finding 85:** Plugging away at the role-making of involved fatherhood [U]

“I feel like….I’m crawling through mud…There is nothing clear….I’m groping.” (7 months post birth)

“Not having time with him I felt a lot of frustration when I had to spend time with him. I just feel like I was incapable and I couldn’t cope. That was the worst feeling that I ever had in my whole life, that I wouldn’t take care of my son when I had to spend time with him because I didn’t know what to do” (7 weeks post birth)^60^^(p.15)^

**Finding 86:** Accomplishing an important goal in this life phase [C]

“I think if you go by the traditional Chinese way of thinking, you’ve reached the point of having a baby, and this is what you should be doing at this point”.^61^^(p.63)^

**Finding 87:** Proving their ability as men [C]

“My first thought was ‘yes! I can have a baby”^61^^(p.63)^

**Finding 88:** Symbolizing eternal love [C]

“I imagine my dad taking both my mom and my child out….My child will make my parents feel very happy. I already imagine what it must be like so I have already begun fostering a good family atmosphere”.^61^^(p.63)^

**Finding 89:** Ending their wives’ discomfort [C]

“I would like to say that soon my wife will not be suffering any longer. She's been through a hard time; before she became pregnant, and now, while she is expecting this baby. As far as I know, she has gone through many hurdles such as examinations and extracting her legs. I’m not even sure if I could do the whole thing once and she tried many times. So, she is a great women….. Now it's successful and she’ll never have to go through any more suffering!”^61^^(p.63)^

**Finding 90:** A different mission and challenge [C]

“I don’t know how to interact with my child when she's born….I’ve never been a father, so I feel quite terrified”.^61^^(p.64)^

“I feel so panicky because I don’t know what to do during the labor and delivery. I have no idea what kinds of situations I am going to meet…..“^61^^(p.64)^

**Finding 91:** The health status of his wife and fetus [C]

“If the baby isn’t healthy, I’ll be worried because I don’t know if it's good for a baby to grow like that”^61^^(p.64)^

**Finding 92:** Discouraged by the inapplicability of the old ways of building relationships [C]

“….My wife can share her feelings with me. Sometimes she says the baby is moving inside her. But, actually, as a third person, I can’t imagine what that's like”.^61^^(p.65)^

“My wife often complains that I don’t care about our unborn girl. I won’t listen to her heartbeat or look at her belly movements at home. The reason is, I don’t know how to do that. It seems strange to me. …I feel it isn’t necessary to do that. …I can’t see the baby so I can’t make-believe all those gestures. When the baby arrives I’ll be able to hold her and play with her, but right now she's not real. May be it's because … I can’t say she doesn’t really exist but she's still not actually real”.^61^^(p.65)^

**Finding 93:** Adjustment [C]

“……She often complains that I download “noise” from the internet. She thinks it's not music. I feel bad when she keeps going on at me about this. I just go outside and have a smoke”^61^^(p.65)^

**Finding 94:** Preparation for fatherhood [C]

“Money is also very important. We therefore have to save as much as we can. I need to work as hard as possible. May be I’ll need some investments as well”.^61^^(p.66)^

**Finding 95:** Engagement [U]

“My heart feels warm when I talk to him…. I feel like it's listening to me seriously and then he looks at me with a pair of curious eyes”.^61^^(p.66)^

**Finding 96:** The wonder of fetal movement [C]

Author: If the fetal movements became strong enough to disturb the mothers’ sleep and cause discomfort, however, some of the expectant fathers became worried that they would hurt their mothers. When these movements happened some of the expectant fathers talked to their unborn infants and asked them not to move so violently.

“I say ‘Baby, be nice, do not move so vigorously. Your mommy might feel the pain”.^61^^(p.67)^

**Finding 97:** Expanded vision [U]

“As for me, life's changed a lot, especially after experiencing this. I mean, giving birth to a new life. This has changed my viewpoint on life enormously. I’ve made great progress here. This small life has influenced my life in so many ways. It has changed the way I look at things, my attitudes and the way I treat people. All this is totally different from the person I used to be”.^61^^(p.68)^

**Finding 98:** Experiences during pregnancy: Feelings of separation [C]

“Well before he was born I held Jenni's [wife's name] tummy, but with the best will in the world, it's just a bump that moves, like something out of a Ridley Scott film [laughs] it's weird and an extraordinary thing, no doubt about it, but I did find it different and I feel that it has to feel different with the mum as they are carrying the baby and feeling it move and grow inside, that must mean that the emotional attachment that must build must be extraordinary and I don’t think that any bloke could ever understand that”^62^^(p.5)^

**Finding 99:** Fatherhood – the early days: Helplessness [U]

“You’re not overly sure what you’re supposed to be doing, and there are times when you have the emotion of complete helplessness.”^62^^(p.6)^

**Finding 100:** Fatherhood – the early days: Trial and error parenting [U]

“Initially it is all about trial and error, at least that's how it was for us, purely trial and error … in the early days we were both sort of saying, what's wrong with him? Is it his nappy? Is it food? Is it sleep? And you go through that sort of list until you find something that makes him quiet and you go, well it was that then, and so you start to notice those signs a little more each time”.^62^^(p.6)^

**Finding 101:** Fatherhood – the early days: She leads, I follow [C]

“I learned a lot from watching Jane [wife] with him, you know how to hold him, change a nappy, bathe him”^62^^(p.6)^

**Finding 102:** Fatherhood – the early days: Working together [C]

“Another thing we did was the both of us were getting up in the night to deal with her [daughter] and we soon realised that maybe I needed some more sleep so Anna [wife] would get up and do all the night feeds one night and I would do all the night feeds the next night …we soon got her onto the bottle so I could help out with the dream feeds while Anna slept and when she got up to do the next feed I would be able to go to sleep … working in partnership is key”.^62^^(p.6)^

**Finding 103:** Fatherhood – the early days: Gaining confidence and regaining control [C]

“It was purely about experience and from that comes confidence … the more you do the more you learn and as time goes on you remember how you’ve dealt with things in the past … I wanted to make sure that I got stuck in … being off work for a month gave me the opportunity to get involved”^62^^(p.7)^

**Finding 104**: Changes associated with the father's Role [U]

“Our lifestyle has changed completely, in ways for the better but it is a massive struggle, it's like taking on another job almost because it has been very tiring, a lot of hard work, a lot of sleepless nights…the further you go back the worse it was…learning everything, being a dad for the first time everything is brand new.”^63^^(p.43)^

**Finding 105:** Bonding and Co-parenting [U]

“I feel like our bond has grown. I think when it started off she was such a responsibility, she was such a … burden is not the word … she was such hard work that I think it is difficult to build a bond straight away…. I think your resentment of “you are making me get up at this time, making me do this again” is quite overpowering but as they get older you play with them more, see their personality … your bond grows.”^63^^(p.43)^

**Finding 106:** Experience of the NHS and father's well-being [U]

“I think the thing that struck me was you are either treated as a couple having a child or as a mother. There is nothing focused on or no support groups for fathers. There is nothing to help you prepare for your role…”^63^^(p.46)^

“The support for fathering has been non-existent. I have happened to be here when health visitors came around but that is coincidental, there is nothing directed at fathers. I suppose you just get on with it…. There isn’t a dad's support network and I am lucky that I haven’t needed it … but if things had been different I think it would have been harder.”^63^^(p.46)^

**Finding 107:** Worklife [U]

“After this last week away and seeing him grow and then going back to work and having 15 minutes a day with him … it has made me realise what I am missing and it is hard because you want to be there and you want to see everything… [The bond] has developed but because I don’t get to see him as often as I would like it is a constant worry that it is not developing how I would want it to… “^63^^(p.50)^

**Finding 108:** Government and Society [U]

“I think the government or society thinks that the father is not always needed at home, that is why a system is created with only 14 days leave. We could have done with more … it appears like the father has to be moved out of the house as soon as possible.”^63^^(p.52)^

**Finding 109:** Struggling between stereotypes and personal perceptions of male sexuality during the transition to fatherhood: Societal view of sexuality [C]

“There is no one who hangs or shoots himself just because he could not make love for some time”.^64^^(p.720)^

However, some participants had an opposing view.^64^^(p.720)^

“I think that men have more sexual needs, they are a little bit hornier”.^64^^(p.720)^

**Finding 110:** Struggling between stereotypes and personal perceptions of male sexuality during the transition to fatherhood: Expectations on sexuality in the relationship after childbirth [U]

“Prior to the birth you think, ‘a few weeks abstinence,’ but now when the child is born…it can be half a year.”^64^^(p.720)^

**Finding 111:** New frames for negotiating sex: Changes in the relation after childbirth [C]

“I think it is important to be there … if the mother and child becomes one unit, it will be too much ‘they’ and you will be left out a bit. Then it will be tough to get it together”. (to become a triad)^64^^(p.720)^

“Altogether, sexual life is important in a relationship. To ‘K’ it comes far down on the priority list. To sleep 10 hours during the night, cleaning the house, doing the laundry and…when all this is done she can start thinking about having sex^64^^(p.721)^

**Finding 112:** New frames for negotiating sex: Experience of sexual life after childbirth [C]

“It is good to try to have sex, maybe not a proper intercourse, but still… “^64^^(p.721)^

“she has got bigger boobs now; they are so full, tender, and awesome”.^64^^(p.721)^

**Finding 113:** New frames for negotiating sex: Physical and mental alterations in partner [C]

“It [the laceration] has influenced our sex life and still does. Her skin has lost sensitivity. It causes her some discomfort. But if you compare it with the loss of desire and the constant fatigue, it is a petty hindrance, really”.^64^^(p.721)^

“She got depressed, she was crying all the time, sex was not on the map, I focussed on and took care of our son”.^64^^(p.721)^

**Finding 114:** A need to feel safe and at ease with sex in the new family Situation: Communication [US]

Authors’ comments: The new fathers had different ideas about what to expect from that visit. The participants said that they thought that it would be important to talk about sex in a relaxed way and not to be stressed about it.^64^^(p.721)^

**Finding 115:** A need to feel safe and at ease with sex in the new family situation: Reassurance [US]

They experienced the visit to be quite focussed on the baby and less on the relationship and on sexuality. The men perceived that it was difficult to get accurate information about the changes in the sexual relationship after childbirth.^64^^(p.721)^

**Finding 116:** Caring for the baby in both health and illness [C]

“Every baby is different. You have to expect the unexpected and not have too many preconceptions about how the baby should behave”.^65^^(p.88)^

**Finding 117:** Breastfeeding: more challenging than expected [U]

“I have to say that there I was not prepared at all but had a mental picture that it's just a matter of laying the baby to the breast and it all works. When it didn’t work you stood there: aha, what the hell do we do now?”^65^^(p.88)^

**Finding 118:** Still being a couple but not as before [C]

“That there's a lot of focus on the child… and the partner in the relationship gets forgotten. And then sexual life…you need to be aware of…you want to know what's normal? It's important that it's still a relationship, but you can adjust the relationship a bit”.^65^^(p.88)^

**Finding 119:** Being tired and bound [C]

“I don’t know how you prepare yourself for sleep problems but…. (laughs)…with sleeplessness comes irritation. It effects… or it can effect the relationship between me and X…^65^^(p.89)^

**Finding 120:** Understanding emotional reactions [C]

“You don’t need to feel this great surge of happiness that everyone writes about on Facebook. I can still feel…yes it's happiness but it's stressful…I still haven’t adjusted to it…”^65^^(p.89)^

**Finding 121:** Adjusting priorities [C]

“You’ve got to leave your juvenile life behind, stop running around with your mates and that. You have to change it…without agreeing to it…you’ve just got no choice (laughs). Now I’ve got someone else to think about…”^65^^(p.89)^

**Finding 122:** Acknowledging ones’ limitations [C]

“You can’t prepare yourself for everything but if this thing happens you have information and the necessary prerequisites to deal with the situation”.^65^^(p.90)^

**Finding 123:** Dealing with internal and external pressures [C]

“It feels like you’re being checked out by those around you…how you get on in different situations…because a lot of people have their views. It's sort of tough…at the same time, you don’t want to do the wrong thing…”^65^^(p.90)^

**Finding 124:** Communicating with ones’ partner [C]

“We’ve talked… through the whole pregnancy because things can change – what you think and believe. That way you avoid irritation and rows”.^65^^(p.89)^

**Finding 125:** Forming a fatherhood identity [C]

“It's important to be prepared for the fact that there will be a lot of mother and baby time. I have to see that they are as comfortable as possible. She's got a full-time job with her (the baby), with breastfeeding, like”.^65^^(p.89)^

“Naturally you have to help out…but it shouldn’t be compulsory that you have to wash-up, shop, do the washing and clean…to relieve the one that's been at home. One has just as much right to be together with one's child…”^65^^(p.89)^

**Finding 126:** Parental groups: the good and the bad [C]

“Parents….get them to ask people who have three kids to join in a discussion… because they’ve already got the gen”.^65^^(p.90)^

“Parental groups are an excellent way to prepare but they were too short… we hardly spoke of the time after birth”.^65^^(p.90)^

**Finding 127:** Internet as an asset or a worrier [C]

“Most of the time on the internet, because … I’m not a patient person. I want to have everything like that (clicks fingers)”.^65^^(p.90)^

“I looked at YouTube, but you don’t know to a hundred per cent which… what experience those showing the film have…Yes, if you think a bit…is it something good or can it be harmful…. ”^65^^(p.90)^

**Finding 128:** The need for guidance [C]

“The midwife was very nice… and she asked: do you have any questions? But you don’t have any questions if you don’t know what is coming. I would know now (after birth) what to ask”^65^^(p.90)^

**Finding 129:** Information: the when and how [C]

“I learn most when someone tells me things….absolutely. So, I prefer that. But it's probably that you need to have a mixture of things….because some learn by reading and seeing.”^65^^(p.90)^

**“**Information needs to be well choreographed, it needs to capture our interest, it needs to be given in a fun way. Use humor: situations can afterwards be looked at as funny or comic but when you are in it, it's like a matter of life or death”.^65^^(p.90)^

**Finding 130:** Emotional changes experienced [C]

“It's… just the best experience in your life! Best experience in your life is when the baby comes out and you see that little, little thing moving there and it's still dirty and trying to look for food and you realise that that is er… that is, your baby, ya. It's amazing. Amazing feelings, you cannot describe”.^66^^(p.783)^

“From the beginning till the end, I never show some unsure, uncertainty. Ya, what I will do and what I should appear to do (laughs!)… I am very confident at her face [in front of her] (laughs).Inside you surely got some nervous (place palm to heart)!”^66^^(p.783–784)^

**Finding 131:** Adaptive and supportive behaviors adopted [C]

“We used to meet on and off during the weekends. So I stopped going there and then even for parties, I used to attend a lot. She can’t stay there for long time. She’ll get pain… So, even if we’re going, we just go and then say hi and spend there 10 min and come back because she can’t stay for more time. Or, if possible, I used to avoid also”.^66^^(p.784)^

**Finding 132:** Social support received [C]

“Generally, it's very useful and supportive if your parents or parents-in-law uuhh… are able to contribute as in, provide advice, share their previous experience and help you to prepare along the way. It's a big encouragement and emotional support ah, from the family”.^66^^(p.784)^

**Finding 133:** Suggestions for improvement to the current maternity care [C]

Provide more information

“But I think… it's much more can do la. Like encourage my wife to do the pregnant lady exercise all these things. Uuhh… for instance, he didn’t really tell me where la”.^66^^(p.785)^

“Another thing is… some of the staff were not that happy to do something, maybe. They just er… do that not very carefully. May be if you ask she… need to do something, she will just do that, just finish, not very carefully to finish”.^66^^(p.785)^

**Finding 134:** Imagining life and needs with a baby: Fantasies and fears [C]

“.. . just encourage them, she is doing well looking after the bub. I think there is often a bit of self-doubt about whether they are doing the right thing and suggest, reassure her you are doing great”.^67^^(p.49)^

**Finding 135:** Imagining life and needs with a baby: Gendered roles [C]

“I mean they [the birth classes] make the father out to be a complete idiot; you are always referred to as the bloke at the end of the bed who got you into the mess in the first place – you know what I mean? Referred to as the guilty party, but you know we’re getting off lightly. We get this little present at the end and we have to do nothing for it, you know”^67^^(p.49)^

**Finding 136:** Imagining life and needs with a baby: Relationships [C]

“.. . now and again you’re probably going to come home and walk in and it's not going to be all champagne and chocolates. You’re going to have bad days and be upset or angry or something and trying to determine and learn the difference between they are not actually angry with you so don’t snap back”.^67^^(p.49–50)^

**Finding 137:** Preferred sources of information and support [C]

“Seeing [on the DVD] not the specialists, not the experts but the guys who were actually going through that situation without knowing much, the way we do. I could identify with those”^67^^(p.50)^

**Finding 138:** The role of primary care in mental health care for new parents: Routine enquiry [C]

Authors interpretation: health professionals’ role should be limited to giving information: ‘they’ve got an obligation to let you know information’ and suspicion that these health professionals are ‘not qualified to emotionally help you’ (M1), because their training prepares them to treat physical not mental illnesses: ‘I don’t know how much of their training would be on the emotional side of things’ (M2).^67^^(p.50–51)^

**Finding 139:** The role of primary care in mental health care for new parents: Screening questionnaires [C]

Men's willingness would depend on how long the form was: ‘where there aren’t too many boxes to tick, three or four… ten's a struggle’ (M4); how they were feeling at the time; ‘the value you think you are going to get from it at the time’ (M4); and whether there were competing priorities.^67^^(p.51)^

**Finding 140:** Feelings of exclusion [C]

“Um things that I find difficult is not being able to stop that, not being able to stop her crying … That's hard because I feel quite helpless you know when she is really screaming her head off. Then Tanya usually has to breastfeed her or sometimes she just likes to nurse on Tanya, on Tanya's breast just to fall off to sleep sort of thing. So that is difficult not being able to do anything about that, I can’t feed her but I can’t do anything”.^68^^(p.21)^

**Finding 141:** Good father and father involvement [C]

“I feel that there is more to come. As Imogen develops I will develop my ability to be a father that will grow and evolve and develop with her”.^68^^(p.21)^

“I’m looking forward to the whole experience of being a father having someone that relies on you, is dependent on you … it's going to be really good to sort of look after it, take care and sort of bring it into the world … ”^68^^(p.23)^

**Finding 142:** Making Active Efforts in Preparation for Childbirth in a Foreign Country [C]

Author: They [expectant fathers] also became more aware and concerned about the changes that they observed taking place in their wives.

“Thinking of my unborn baby, it was very important to focus on my wife's health. This was a natural way of thinking, wasn’t it? I picked up natural foods and pure drinks for my wife for the first time”.^69^^(p.43)^

**Finding 143:** Challenges in Pregnancy, Childbirth, and Parenting as Husbands/Partners [C]

“For men, we don’t feel any pain and don’t experience the difficulty of labor. Thus, we are apt to become less involved with pregnancy and childbirth. We also only need to observe how her body will change because we do not have to experience it ourselves”.^69^^(p.43)^

**Finding 144:** Challenges in Transition to Parenthood [C]

“I think that the relationship between a baby and a father is not a close bond. I can’t feel any fetal movements myself. I also can’t breastfeed my baby. That's why I would not have any connection with my baby if I did not take care of her. The more I take care of my baby, the more I feel like a father”.^69^^(p.43)^
